# Genomic prediction of agronomic traits in perennial ryegrass (*Lolium perenne* L.) and genotype x environment interactions at the limit of the species distribution

**DOI:** 10.1007/s00122-025-05064-x

**Published:** 2025-10-25

**Authors:** Natasha H. Johansen, Andrea Bellucci, Pernille B. Hansen, Petter Marum, Helga Amdahl, Kristin H. Gylstrøm, Odd Arne Rognli, Vilma Kemešytė, Gintaras Brazauskas, Morten Greve, Christer Persson, Mika Isolahti, Áslaug Helgadóttir, Rene Aavola, Torben Asp, Guillaume P. Ramstein

**Affiliations:** 1 Center for Quantitative Genetics and Genomics, Aarhus University, Aarhus, Denmark; 2 Carlsberg Research Laboratory, Copenhagen, Denmark; 3 Nordic Seed A/S, Odder, Denmark; 4 Graminor, Ridabu, Norway; 5 Norwegian University of Life Sciences, Ås, Norway; 6 Institute of Agriculture, Lithuanian Research Centre for Agriculture and Forestry, Akademija, Lithuania; 7 DLF Seeds A/S, Roskilde, Denmark; 8 Lantmännen Seed, Malmö, Sweden; 9 Boreal Plant Breeding, Jokioinen, Finland; 10 The Agricultural University of Iceland, Hvanneyri, Iceland; 11 Centre of Estonian Rural Research and Knowledge, Jõgeva, Estonia

## Abstract

**Key Message:**

Perennial ryegrass shows extensive genotype x environment interactions at the limit of its ecological niche. Accounting for GxE may improve prediction even when environmental and genetic samples are highly diverse.

**Background:**

In breeding the aim is to identify and accumulate beneficial variants. However, detection of these variants may be challenging in the presence of extensive genotype x environment interactions (GxE).

**Methods:**

The study assesses the performance of 264 diploid perennial ryegrass accessions in a multi-environment field trial. We investigate the extent of GxE, for yield (total dry matter) and persistence traits under environmental conditions experienced in Nordic and Baltic regions at the limit of the species distribution. Two different approaches to modelling GxE were tested and validated under three different breeding scenarios.

**Results:**

Our analysis documented the presence of significant GxE for all traits. Validation showed improvements in prediction accuracy when accounting for GxE: up to 4% for yield when predicting in unobserved environments, and up to 22% and 9% for spring cover and winter kill, respectively, when predicting unobserved germplasm. Genome-wide-association-studies (GWAS) were utilized to detect genetic variants with marginal effects (environment-independent effect) and conditional effects (environment-dependent effects). Results showed the presence of large-effect genetic variants with marginal effects, in addition to few Quantitative Trait Loci (QTL) whose effects were adaptive under specific environmental conditions while neutral or deleterious under different environmental conditions.

**Conclusion:**

This study demonstrates the usefulness and limitations of genomic prediction models for predicting GxE in highly diverse samples and describes the extent of GxE at the limit of species distribution for perennial ryegrass. Our study points towards adaptive variation which may enhance persistence of perennial ryegrass populations in Nordic and Baltic growing conditions.

**Supplementary Information:**

The online version contains supplementary material available at 10.1007/s00122-025-05064-x.

## Introduction

Climate changes will in the future lead to increased fluctuations in temperature and rainfall and therefore more unpredictable weather patterns, which may challenge plant breeders in the future (Raza et al. 2019). Consequently, to ensure that future food demand can be supported, it is necessary to adapt current food production practices and develop high yielding crop varieties that are adapted to future environments (Kahiluoto et al. 2019). Different selection strategies have been developed to facilitate efficient evaluation of superior breeding material. These selection strategies include, among other, genomic prediction (GP) (Meuwissen et al. 2001) which has become an important tool to predict the genetic performance, i.e. breeding values, of breeding material from genetic markers. Generally, plant breeders evaluate candidate lines or populations in multi-environment trials (METs) to identify broadly adapted genotypes that consistently thrive and yield well across a range of intended production environments, collectively referred to as the target population of environments (TPE). However, broad adaptation does not necessarily imply superior performance across environments, as some lines/populations may express enhanced performance in a narrow range of environments, due to local adaptation to specific environmental conditions (Ågren and Schemske 2012; Ågren et al. 2013; Lasky et al. 2017; Blanco-Pastor et al. 2021). Differences in phenotypic performance between lines/populations are not exclusively caused by genetic effects but are also affected by genotype by environment interactions (GxE), i.e. differential responses among genotypes to a change in environment. GxE can be expressed as (i) non-cross-over interaction, i.e. genotypes maintain their rankings. (ii) cross-over interaction where the rankings of the genotypes differ across environments (El-Soda et al. 2014). Traditionally, plant breeders have ignored or minimised GxE to avoid the challenge of modelling this component, however, studies show that the prediction accuracy of GP models can be enhanced in MET, by accounting for GxE (Jarquín et al. 2014, 2017; Cuevas et al. 2017; Millet et al. 2019; Jarquin et al. 2020). Furthermore, integration of GxE in GP models allows for prediction of phenotypic performance in unobserved environments, which can be of advantage to breeders. Different approaches have been developed to integrate GxE into genomic prediction models. These include amongst others the reaction norm (Jarquín et al. 2014) and envirotyping approaches (Xu 2016; Costa-Neto et al. 2021a, b, c). These approaches differ regarding how the environmental covariates are transformed. The reaction norm approach models GxE by genotypes’ linear response to environmental covariates. Thus, in reaction norm models, more complex interactions, i.e. nonlinear interactions, are not captured (Jarquín et al. 2014). Alternatively, envirotyping is an approach by which environments are characterised by determining the environmental covariates that affect a given plant species’ growth (Xu 2016; Costa-Neto et al. 2021a, b, c). The envirotyping approach is comparable to the reaction norm approach but differs in how environmental relatedness is calculated. In the reaction norm approach, environmental relatedness is typically based on quantitative descriptors (e.g. mean temperature). In contrast, the envirotyping approach partitions environmental gradients into environmental zones, or typologies, based on physiological expectations related to survival, tolerance, or optimal growth for the given crop species, as inspired by Shelford’s Law of Tolerance (Shelford 1931). Specifically, environmental zones are defined for each of the pre-selected environmental variables, allowing for the frequency of different zones/typologies to be calculated for each environmental covariate x environment combination. This enables environmental relatedness to be quantified based on the frequency of each of the defined environmental zones experienced by an environment. The envirotyping approach therefore differs from the reaction norm approach in that it also informs about the value of the environment for the given crop (Costa-Neto et al. 2021a, b, c). The aim of GP is to identify superior lines based on genome-wide variants; however, GP cannot be used to detect impactful variants at a resolution that allows for detection of potential QTL (Quantitative Trait Loci). Instead, phenotype-genotype associations are identified using Genome-Wide-Association-studies (GWAS) (Liu and Yan 2019; Tibbs Cortes et al. 2021). Linkage disequilibrium (LD), i.e. non-random association of alleles at different loci, is integral for GWAS as strong and tight linkage between causal and noncausal alleles at proximal loci increases the likelihood of detecting significant phenotype-genotype associations, even when the causal variant is not genotyped (Flint-Garcia et al. 2003). Thus, association analysis in populations characterised by rapid LD decay requires high marker densities, as genetic variants will be linked together in smaller blocks, compared to populations with more extensive linkage. GWASs are commonly used to detect QTL based on alleles’ average association with traits, also termed marginal effect, but may also be used for detection of variants with conditional effects, i.e. environment-dependent effects (Moore et al. 2019; Li et al. 2021). Perennial ryegrass (*Lolium perenne* L*.)* is an economically important forage grass used for animal feed in dairy production, where it is valued because of its high yields and nutritional value. Additionally, the crop expresses a high level of genetic diversity, due to its obligate outbreeding nature (Brazauskas et al. 2011). Nordic and Baltic regions, which have previously been unsuitable for production of perennial ryegrass, can become fit for production of certain robust and cold-tolerant varieties in the near future as temperatures increase in these regions in response to climate changes (Ergon et al. 2018; Helgadóttir et al. 2018). This has created an interest, amongst commercial breeders of perennial ryegrass, in extending the production of this crop further North (Solberg et al. 1994; Helgadóttir et al. 2018). Previous experimental work suggests significant levels of GxE in perennial ryegrass (Grogan and Gilliland 2011). These GxE interactions include rank changes, i.e. environment-specific ranking of accessions in respect to productivity (Grogan and Gilliland 2011) and large GxE at year and field level (Fè et al. 2016).

However, given that the Nordic and Baltic countries span a wide range of environmental conditions, we may expect more marked GxE than observed in previous studies focussing on more homogenous environments. Thus, greater insight into the extent of GxE, in perennial ryegrass, in Nordic and Baltic regions may benefit and support future breeding efforts towards more robust perennial ryegrass germplasm.

Few studies have previously investigated GxE in perennial ryegrass and general questions remain about the performance of GxE models in diverse samples as investigated in the current study, which includes both natural and breeding populations. Thus, taken together, the diverse environmental conditions experienced in Nordic and Baltic regions, in addition to the diverse genetic background of perennial ryegrass, arising from its extensive genetic diversity and the limited gene flow between natural populations (Blanco-Pastor et al. 2019), may contribute to pronounced GxE.

Hence, this study aims to: (i) investigate and assess the performance of genetically diverse perennial ryegrass accessions in a multi-environment field trial, to improve biomass yield and persistence traits, (ii) determine the extent of GxE in perennial ryegrass to assess their potential for adaptation to extreme environments at the margin of the species’ distribution, and (iii) detect adaptive variants which confer significant advantages either across the tested environments, or under specific environmental conditions.

## Materials and methods

### Plant material and phenotyping

A total of 264 diploid perennial ryegrass accessions were included in this study. These accessions consisted of advanced cultivars, wild/semi-wild accessions, and landraces obtained from gene banks worldwide, including the Nordic Genetic Resource Center (Rognli et al. 2018). Accessions were multiplied to obtain enough seeds for regular field trials. For the study, a diverse set of accessions was chosen, in respect to geographic origin, which spanned Central Europe, Northern Europe, Russia, US and Japan (Table S1). The experiment was conducted across 2014–2016 in Norway (60°45’ N, 11°12’ E), Estonia (58°45’ N, 26°24’ E), Sweden (55°56’ N, 13°6’ E), Lithuania (55°23’ N, 23°52’ E), Iceland (64°9’ N, 21°45’ W) and Denmark (55°20’ N, 12°23’ E). Replicated field trials were established in late-spring or summer in 2014, in Norway (20th of June), Estonia (2nd of August), Sweden (28th of May), Lithuania (10th of June), Iceland (9th of July), and Denmark (25th of June). Plot sizes varied by location; Denmark (12.5 m^2^), Estonia (7.6 m^2^), Iceland (5 m^2^), Lithuania (8.25 m^2^), Norway (6 m^2^), and Sweden (8.8 m^2^). The elevation, in metres above sea level, or m.a.sl, at the trial sites were as follows: Norway (187 m.a.sl), Estonia (68 m.a.sl), Sweden (65 m.a.sl), Lithuania (33 m.a.sl), Iceland (35 m.a.sl) and Denmark (35 m.a.sl). The following traits were measured in the study: total dry matter yield from three harvests during the growing season (TDM3), spring cover (SpringCover), winter kill (WinterKill). The traits SpringCover and WinterKill are transformed to a scale ranging from 1 to 9, where SpringCover, i.e. percentage of plot covered by the crop, is a measure of the crop’s establishment ability, while WinterKill is measured as the decrease in plot cover from autumn to spring, with 1 for highest survival, 9 for lowest. The data was unbalanced with respect to the number of accessions scored in each country, with the number of accessions varying between 108 and 262 depending on the environment, while across all environments a total of 264 accessions were included. Two replicates were included for each accession in the environments wherein the accessions were tested. Hence, of the 264 accessions included in the study, a ‘core’ set of 108 accessions were evaluated in all countries. However, due to environmental differences, not all traits were scored across all countries, i.e. WinterKill was not assessed in environments which did not experience stressful winter climates, while TDM3 was not scored in Iceland because the environmental conditions limited the experiment to only two harvests for that location, limiting the comparability of yield production in this environment to the other environments, where yield (TDM3) was harvested at least three times a year.

### Genotyping-by-sequencing

Sequence data were produced using genotyping-by-sequencing (Elshire et al. 2011), with the methylation-sensitive restriction enzyme ApeKI to target the low copy fraction of the genome. Sampling and library preparation followed the protocol described by Byrne et al. (2013). The accessions were genotyped based on a pooled sample. After alignment of sequencing reads to the reference genome (Nagy et al. 2022) and SNP calling by GATK (McKenna et al. 2010), a total of 380,252 SNPs were obtained. VCF-tools (Danecek et al. 2011) were used to filter the SNP markers that did not have sufficient coverage, i.e. Depth (DP) referring to the number of sequencing reads at a specific genomic position. Following requirements were used: minDP $\ge$ 10, min-mean DP $\ge$ 20, max-meanDP $\ge$ 100, minQ $\ge$ 30, minor allele frequency across accessions (MAF) $\ge$ 0.05, missing rate lower than 20%. After filtering, 151,074 SNPs remained. We then calculated the allele frequency, in each accession *i* where pseudo-counts were used to reduce bias introduced by low “read depths”: ${p}_{ik}= \frac{{\text{AD}}_{\text{ALT},ik}+1 }{{\text{AD}}_{\text{ALT},ik}+{\text{AD}}_{\text{REF},ik}+2}$, where ${\text{AD}}_{\text{REF},ik}$ and ${\text{AD}}_{\text{ALT},ik}$ are the depths of the alternate and reference alleles, respectively, in accession *i* at marker *k*. Missing information at a given SNP was imputed as the average allele frequency across accessions.

### Genomic relationship matrices

To construct the covariance matrix for additive effects the genomic relationship matrix (GRM) was constructed as ${\mathbf{G}}_{A}= \frac{\mathbf{X}{\mathbf{X}}^{\mathbf{T}}}{tr(\mathbf{X}{\mathbf{X}}^{\mathbf{T}})/n}$ (Vitezica et al. 2017), where **X** is the centred matrix of alternate allele frequency matrix for each accession and marker, *tr* is the sum of the matrix diagonals, and *n* the number of accessions. To construct the covariance matrix for dominance effects, we assumed Hardy–Weinberg equilibrium (HWE) in the populations, and estimated marker-specific heterozygosity as ${H}_{ik }=2{p}_{ik }{q}_{ik}$, where ${p}_{ik}$ is the frequency of the alternative allele, and ${q}_{ik}$ is the frequency of the reference allele in accession *i* at marker *k*. Dominance codes were computed by regressing expected heterozygosity, under HWE, on observed allele frequency, and then extracting the model residuals. By using the residuals we effectively decorrelate additive and dominance codes, thus reducing collinearity between additive and dominance model components in downstream models. The computed dominance codes were used to construct the dominance genomic relationship matrix ${(\mathbf{G}}_{D})$ following the approach described for ${(\mathbf{G}}_{A})$. The approach described above is an extension of the method presented in (Alvarez-Castro and Carlborg 2007; Vitezica et al. 2017). Consequently, the dominance model components should capture genetic variation orthogonal to the additive components (dominance deviations), which correlate with expected heterozygosity under HWE.

### Environmental data and environmental covariates

Daily weather and climatic variables were extracted for the six trial locations in the period 2014–2016. The information was collected with the R package EnvRtype (Costa-Neto et al. 2021c). Different environmental covariance matrices are constructed for two GxE modelling approaches: (i) reaction norm (RN) and (ii) envirotyping (ET). Environmental similarity was determined based on 18 environmental covariates. For the RN approach we determined the environment means for each environmental covariate x time-window combination. For the ET approach, daily measurements for each of the environmental covariates were partitioned into quantile intervals (0.05, 0.25, 0.50, 0.95) specific to each time window, intended to characterise environmental zones or envirotypes see Supplementary Table S2. The frequency of each envirotype was then determined for each country x year x time-interval combination. For TDM3, the environmental covariates were extracted from the period of the 1st of September, in the year prior to harvest, to the 31st of August the following year (harvest year). For SpringCover and WinterKill, environmental covariates were extracted from the 1st of September in the year prior to when the trait was scored until the 31st of May (year trait is scored). The R-package EnvRtype requires the specification of lower and upper temperature bounds for crop development for the model crop, to which we used experimentally determined temperature bounds as estimated in (Monks et al. 2009). Specifically, the lower temperature bound was specified to 3.2 °C, lower optimum temperature to 10 °C, and upper optimum temperature to 30 °C, while the upper temperature bound was specified to 52 °C. In both approaches the time interval was specified to span a 30-day window. The covariance matrices, denoted by **E**, were computed with the same approach as used for the genomic relationship matrices. For the RN and ET models, we defined environments as country-year combinations, hence 12 environments were included in the study (Table 1).

**Table 1:** Number of diploid accessions scored in each country for the traits

	Norway		Estonia		Sweden		Lithuania		Iceland		Denmark	
	2015	2016	2015	2016	2015	2016	2015	2016	2015	2016	2015	2016
TDM3	213	213	144	144	108	108	118	118	–	–	262	262
SpringCover	213	213	144	144	–	–	118	118	142	142	–	–
WinterKill	–	–	–	144	–	108	-	118	–	–	–	–

### Phenotypic analysis

Linear mixed models were fitted separately in each location to estimate the effects of accession and their interaction with year. Phenotypic records were regressed on accession (fixed), year (random), the accession-by-year interactions (random), spatial effects nested in year (random), and model residuals. The effects of year, the effects of accession-by-year interactions, and model residuals were modelled as independent and identically distributed (*i.i.d.*), following a normal distribution with the same variance for each type of random effect. Random spatial effects differed by countries, due to differences in experimental design: in Denmark, effects of trial and effects of row and column across the field; in Iceland and Lithuania, effects of replicate and effects of row and column in each replicate; in Estonia, Norway and Sweden, effects of trial and effects of row and column in each trial. Effects of trials and replicates were modelled as *i.i.d.*, whereas effects of rows and columns were modelled as normally distributed following a first-order autoregressive correlation structure, by row and column. In each year, accession means were calculated as the sum of the estimated effects of accession and accession-by-year interactions. Due to differences in management practices across countries, the adjusted accession means, after accounting for micro-environmental effects, were centred and scaled (z-scaling) in each country x year combination, based on the mean and standard deviation estimated in the core collection of 108 accessions (to account for the unbalanced number of accessions by country) (Fig. S1). Z-scaling of the accessions means (within each country-year combination) will contribute to adjusting for management effects in addition to reducing GxE due to scale changes, i.e. GxE due to changes in phenotypic variance between environments which may arise due to differences in both management and climatic conditions.

### Genomic models

#### Main effects of genotype and environment (M1)

Genomic Best Linear Unbiased Prediction models (GBLUP) (Habier et al. 2007) were used, where the simplest model, M1, accounts for the main effect of genotype and environment.

$${y}_{ij}=\mu +{a}_{i}+{e}_{j}+ {\varepsilon }_{ij}$$ (1)

where ${y}_{ij}$ is the estimated mean phenotypic performance for accession $i$ in environment $j$ (country-year combination); ${e}_{j}$ the random effect of environment *j* $\mathbf{e}\sim {\rm N}\left(0, { \sigma }_{E}^{2}\mathbf{E}\right)$; $\mu$ is the intercept, or grand mean; ${a}_{i}$ is the random additive genetic effect of accession *i*, $\mathbf{a}\sim {\rm N}\left(0, { \sigma }_{{G}_{A}}^{2}{\mathbf{G}}_{A}\right);$ while ${\varepsilon }_{ij}$ is the error term, ${\varvec{\varepsilon}}\sim {\rm N}\left(0, {\sigma }_{ \varepsilon }^{2}\mathbf{I}\right)$, where **I** is the identity matrix.

#### Additive genetic effect and environment interaction (M2)

The M1 model is extended by adding the GxE term, which is calculated by the Kronecker product of the covariance for the accessions and the covariance for the environments, given as ${\mathbf{G}}_{A}\otimes \mathbf{E}$, where $\mathbf{ae} \sim \mathrm{N} \left( 0, \mathbf{G}_A \otimes \mathbf{E} \, \sigma^2_{G_{A \times E}} \right)$;

$${y}_{ij}=\mu +{a}_{i}+{e}_{j}+ a{e}_{ij}+ {\varepsilon }_{ij}$$ (2)

#### Additive and dominance genetic effects and their interaction with the environment (M3)

In model M3 we added the random genetic effect of dominance *d*_i_ and its interaction with the environment, where $\mathbf{d}\sim \mathbf{\rm N}(0, {{\mathbf{G}}_{D}\sigma }_{{G}_{D}}^{2})$ and $\mathbf{de} \sim \mathrm{N} \left( 0, \mathbf{G}_D \otimes \mathbf{E} \, \sigma^2_{G_{D \times E}} \right)$.

$${y}_{ij}=\mu +{a}_{i}+{e}_{j}+ a{e}_{ij}+ {d}_{i}+ d{e}_{ij}+ {\varepsilon }_{ij}$$ (3)

#### Heritability

Narrow-sense heritability (${h}^{2}$) measures the proportion of the phenotypic variance which can be explained by the additive genetic variance: Narrow-sense heritability was calculated based on the variance components from the M3 model as:

$${h}^{2} = \frac{{V}_{GA}}{{V}_{P}}=\frac{{\sigma }_{GA}^{2}}{{\sigma }_{GA}^{2} +{ \sigma }_{GD}^{2} + {\sigma }_{GAxE}^{2}+ {\sigma }_{GDxE}^{2} + {\sigma }_{\varepsilon }^{2}}$$

#### Linkage disequilibrium

Linkage-disequilibrium (LD) was estimated by determining the pairwise squared Pearson correlation (r^2^) between markers located on the same chromosome. Correlation was estimated based on the allele frequency of the alternative allele in the different accessions.

### Model comparison and prediction accuracy

Likelihood ratio tests based on restricted maximum likelihoods (REML) were used for model comparison. The likelihood ratios were calculated as: $LR= -2 \left[{l}_{0}\left(\theta \right)-{l}_{1}\left(\theta \right)\right]$, where ${l}_{1}\left(\theta \right)$ is the log-likelihood of the tested model, and ${l}_{0}\left(\theta \right)$ the log-likelihood of the null model. The likelihood ratio test statistic follows a chi-square distribution, where the degrees of freedom is the difference in the number of parameters (random effects) between the models. The models were tested under three different validation schemes (i) Leave-one-country-out, where the models’ ability to extrapolate to unobserved environments was tested. (ii) Leave-one-random-cluster-out, where accessions are randomly assigned to ten clusters. The validation scheme is used to evaluate model ability to predict unobserved accessions when related genetic material is included in the training-set. (iii) Leave-one-genetic-cluster-out, where we divided accessions into 10 genetic clusters based on genetic similarity. This validation scheme is used to evaluate model performance when the genetic similarity between accessions in the training and test set is low. Genetic clusters were inferred by hierarchical clustering based on Ward’s criterion on the matrix of alternate allele frequencies **X**, with Euclidean distance as distance metric. Prediction accuracy was calculated for each fold as the Pearson correlation between observed accession means and predicted accession means ${\widehat{y}}_{ij}$: $\text{Cor}({y}_{ij}, {\widehat{y}}_{ij})$. Finally, model performance was calculated as the mean correlation across all folds within the given validation scheme.

### Genome-wide-association study (GWAS)

A linear mixed model approach was applied to identify associations between accession means and genetic variants. Two different GWAS models were fitted: (i) a ‘marginal’ GWAS model to detect QTL with consistent effects in the suite of environments included in the study; and (ii) a ‘conditional’ GWAS to discover environment-specific QTL effects. To account for population structure, we included the fixed effects of the first three principal components from a principal component analysis (PCA) of the centred allele frequency matrix **X**. In marginal GWAS, the fixed effect of the tested SNP was ${s}_{i}\alpha$, where ${s}_{i}$ is the alternate allele frequency at the tested SNP for accession *i* and *α* is the effect of the alternate allele on the phenotype:

$${y}_{ijk}= \mu + {\mathbf{q}}^{\mathbf{^{\prime}}}{\varvec{\upbeta}}+ {s}_{i}\alpha +{a}_{i}+{d}_{i}{ + c}_{j}+{ac}_{ij}+{dc}_{ij}+ {e}_{jk}+ {\varepsilon }_{ijk}$$ **(Marginal GWAS)**

where **q** is the vector of the principal components for accession *i* and **β** is the vector of associated effects; ${c}_{j}$ is the fixed effect of country *j*; ${a}_{i}$ and ${d}_{i}$ are the additive and dominance genetic effects as described above; ${ac}_{ij}$ and ${dc}_{ij}$ are interactions between genetic effects and country, $\mathbf{ac} \sim \mathrm{N}\left( 0, \mathbf{G}_A \otimes \mathbf{I} \, \sigma^2_{G_{A \times C}} \right)$ and $\mathbf{dc} \sim \mathrm{N}\left( 0, \mathbf{G}_D \otimes \mathbf{I} \, \sigma^2_{G_{D \times C}} \right)$; ${e}_{jk}$ is the random effect of environment *jk* (combination of country *j* and year *k*). This term was excluded for traits scored in a single year. Finally,${\varepsilon }_{ijk}$ is the model residual.

To detect genetic variants in linkage with QTL which have environment-specific effects, we extended the marginal GWAS model by including the interaction between SNP and country:

$${y}_{ijk}={\mu +\mathbf{q}}^{\mathbf{^{\prime}}}{\varvec{\upbeta}}+ {s}_{i}\left(\alpha +{\delta }_{j}\right)+{a}_{i}+{d}_{i}+ {c}_{j}+{ac}_{ij}+{dc}_{ij}+ {e}_{jk}+ {\varepsilon }_{ijk}$$ **(Conditional GWAS)**

where ${\delta }_{j}$ is effect of the SNP in country $j$. Significance was assessed by a Wald test. To correct for multiple testing, statistical significance was assessed by the false discovery rate (FDR) (Benjamini and Hochberg 1995).

### Software

All models were constructed in R 4.2.0** (**Development Core Team**)** within high-performance computing cluster GenomeDK. The R-package MM4LMM version 3.0.2 (Laporte et al. 2022) was used to extract model likelihoods, model validation and to run the GWAS analysis. The R-package EnvRtype version 1.1.1 (Costa-Neto et al. 2021c) was used to extract environmental covariates and construct the environmental covariance matrices for the two modelling approaches. For the phenotypic analysis, we used the software ASREML-R version 4.2 (Butler et al. 2023). The R-package Dendextend was used to infer genetic clusters (Galili 2015).

## Results

### Perennial ryegrass accessions are highly diverse and exhibit minimal population structure

**Fig. 1:**
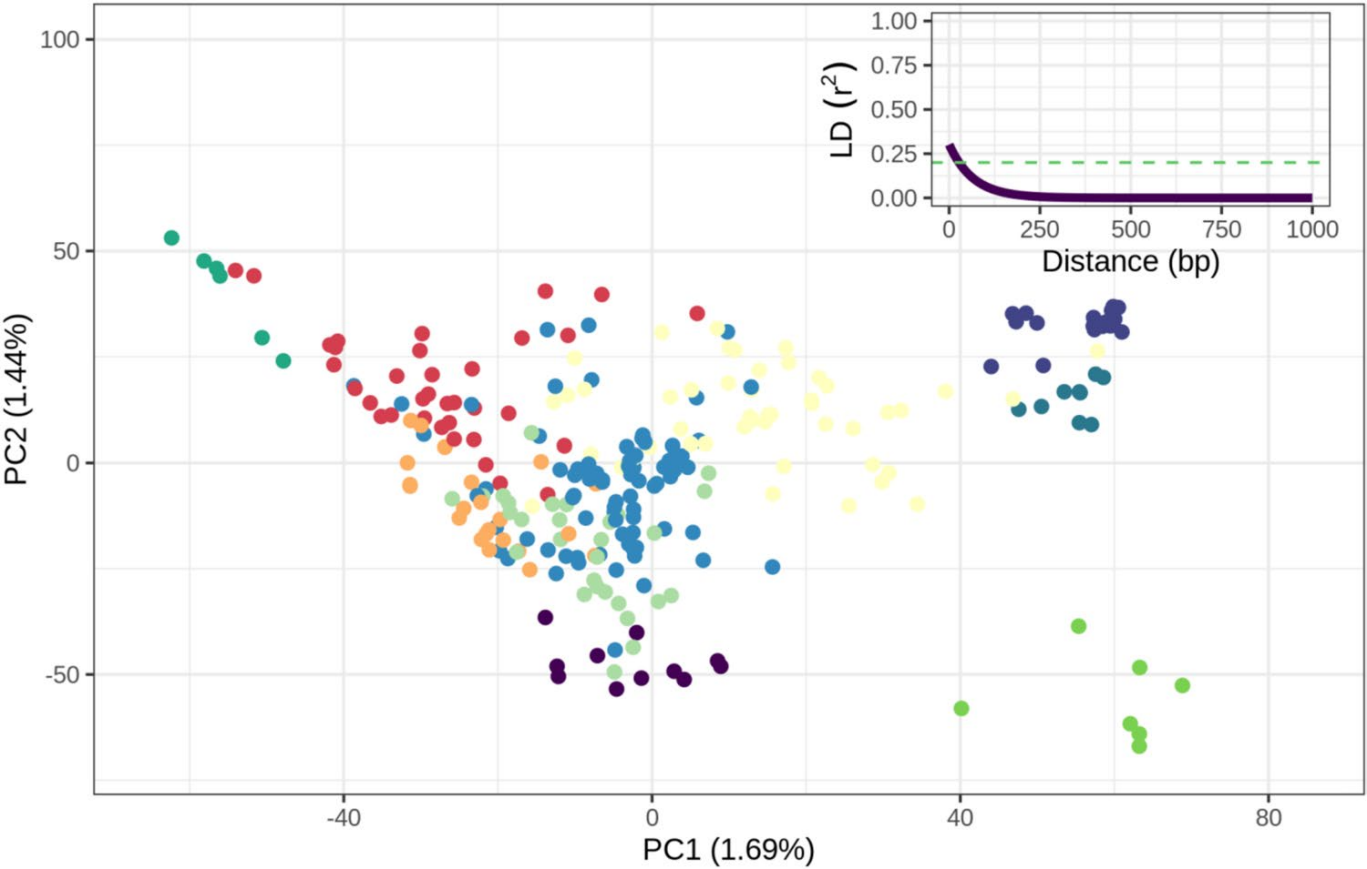
PCA plot of the 264 perennial ryegrass accessions based on their additive allele dosage. Colours indicate which genetic cluster the accessions have been assigned, based on hierarchical clustering. Linkage disequilibrium (LD) decay is shown in the upper-right corner, as a function of distance in pairs (bp). Threshold shown at LD, ${r}^{2}$ = 0.20 (Fig. S2) for relationship between geographic origin and genetic cluster assignment

Generally, the germplasm included in this study was quite diverse, with the first PC accounting for less than 2% of genomic variation. Additionally, we observed rapid LD-decay, with the genome-wide average LD decreasing to below ${r}^{2}<0.2$ within 200 bp (Fig. 1). This is indicative of a germplasm being highly genetically dissimilar, and that alleles at loci in close physical proximity are largely randomly associated. There was a moderate correlation (0.35) between the additive and dominance genomic relationship matrices for the off-diagonal elements, which indicates that there is some collinearity between the additive and the dominance effects. We identified 10 genetic clusters based on hierarchical clustering, which were used for the leave-one-genetic-cluster-out validation scheme (Fig. 1).

### Observed differences among accessions depend on additive and dominance genetic effects and their interactions with climate

**Fig. 2:**
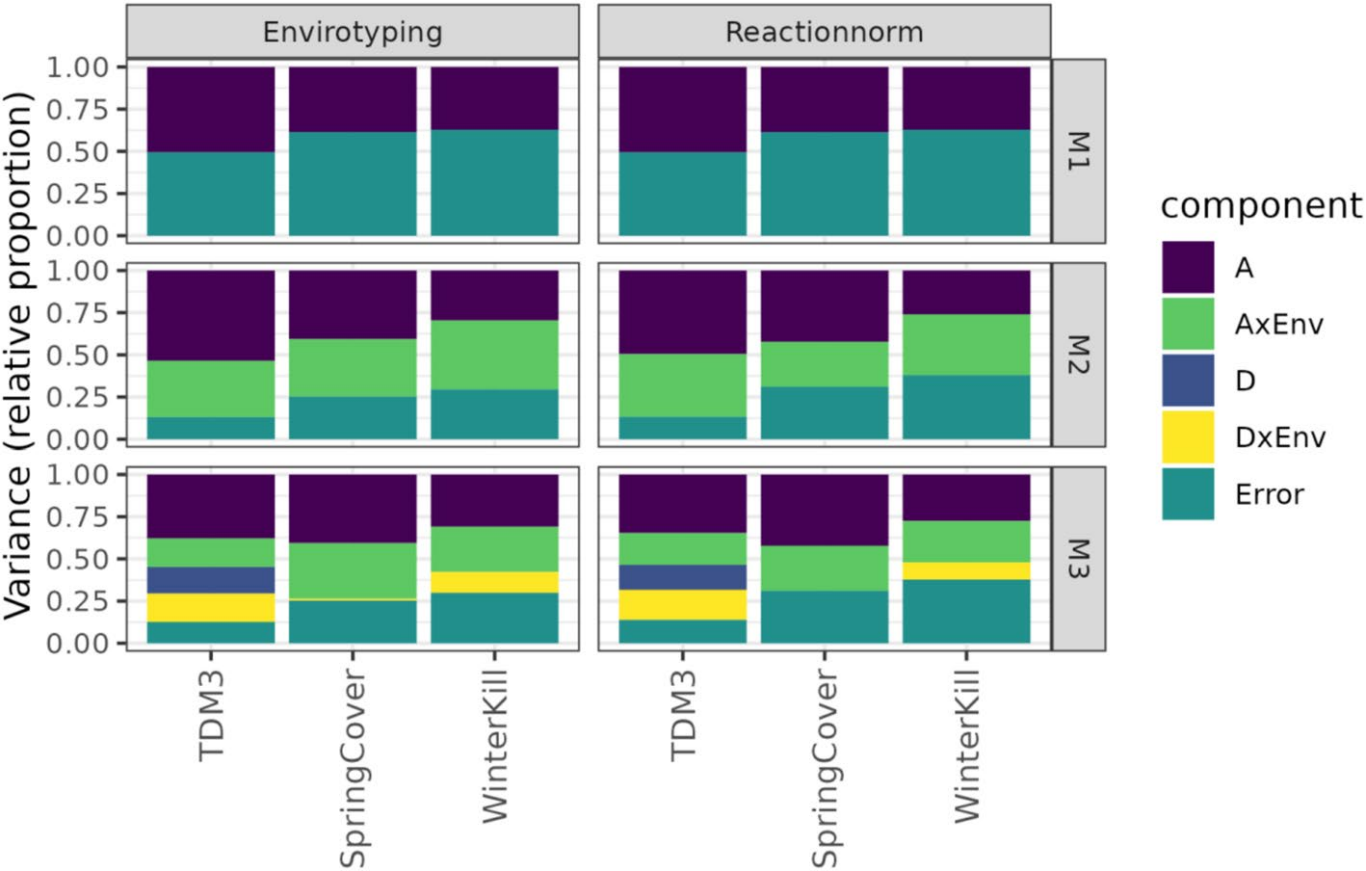
Relative proportion of variance explained by the different model components, excluding the environmental component.** M1**: main effects of genotype and environment; **M2**: main effects of genotype (additive) and their interactions with environment; **M3**: main effects of additive and dominance genetic effects and their interactions with the environment. A: additive genetic variance component, AxEnv: variance component for the interaction between additive genetic effect and environment, D: dominance genetic variance component, DxEnv: variance component for the interaction between the dominance genetic effect and the environment, where Error is the variance component for the residuals

Estimated variance components indicate that the additive genetic components contributed most to the phenotypic variance, regardless of trait or modelling approach (Fig. 2). For the most complex model, M3, the additive genetic component accounted for 34–38% of the variance for TDM3, 41–43% for SpringCover and 27–31% for WinterKill (Fig. 2). For TDM3, the interaction between the dominance genetic effect and environment (DxEnv) constituted about 17-18% of phenotypic variance, while the interaction between the additive genetic effect and environment (AxEnv) constituted about 17–19%. Thus, both forms of GxE contributed approximately equally to phenotypic variance in TDM3. This indicates that heterozygosity, and in turn genetic variation within accession, may be important for environment-specific interactions and local adaptation. For SpringCover and WinterKill GxE are better captured by the interaction between the additive genetic component and the environment (AxEnv), with this component accounting for 27–33% of the phenotypic variance in SpringCover and 25–27% in WinterKill, whereas the interaction between the dominance component and the environment (DxEnv) contributed with 0.4–2% of the variance in SpringCover and 11–12% in WinterKill. The dominance genetic component’s contribution to phenotypic variance was negligible in SpringCover and WinterKill, while in TDM3 it accounted for approx. 15% of the phenotypic variance. The environmental component contributed minimally to the phenotypic variance for all traits, as accession means were centred and scaled in each environment (based on means and standard deviations in the core collection of 108 accessions), to avoid confounding by management effects. Lastly, heritability estimates did not differ markedly between the RN or ET modelling approach for any of the traits, with all traits showing moderate narrow-sense heritabilities (Table 2).

**Table 2:** Narrow-sense heritability (h^2^) for all traits

	TDM3	SpringCover	WinterKill
RN	0.34	0.42	0.27
ET	0.38	0.41	0.31

Heritability estimated with M3 (A + Env + D + AxEnv + DxEnv) for the reaction norm (RN) and envirotyping (ET) approaches

We compared the fits of the nested models M1, M2 and M3 (Table 3). Results show that the addition of an interaction term between the additive genetic component and environment significantly improves model fit for all traits. The addition of a dominance term and an interaction term between the dominance effect and the environment, led to significant model improvement for TDM3, but not for SpringCover and WinterKill. These results suggest the presence of QTL with environment-specific effects which may confer adaptation to specific climatic conditions. Additionally, results indicate that environment-specific marker effects on TDM3 may be mediated by dominance or genetic diversity in addition to additive genetic effects. In contrast, for SpringCover and WinterKill, results indicate that environment-specific marker effects are mainly mediated by additive genetic effects, and the interaction between additive genetic effects and environment. Independent of the trait, we find that the RN and ET models do not differ in their ability to capture GxE.

**Table 3:** Loglikelihood ratio statistics and Pvalues from likelihood-ratio tests used for model comparison

		TDM3	SpringCover	WinterKill
RN	M1-M2	$\text{LLR}= 434.7, P\text{val}= 1.52 \cdot {10}^{-96}$	$\text{LLR}= 180.8, P\text{val }=3.21 \cdot {10}^{-41}$	$\text{LLR} = 9.2, P\text{val }= 2.37 \cdot {10}^{-3}$
	M2-M3	$\text{LLR}= 23.3, P\text{val}=8.09 \cdot {10}^{-6}$	$\text{LLR}=-0.05, P\text{val }=1$	$\text{LLR}= 0.97, P\text{val }=0.615$
ET	M1-M2	$\text{LLR}= 478.5, P\text{val}= 4.61 \cdot {10}^{-106}$	$\text{LLR} = 205.3, P\text{val }= 1.44 \cdot {10}^{-46}$	$LLR = 14.8, P\text{val }= 1.20 \cdot {10}^{-4}$
	M2-M3	$\text{LLR}= 24.2, P\text{val}=5.53 \cdot {10}^{-6}$	$\text{LLR}= -0.03, P\text{val }=1$	$LLR=1.4, P\text{val }=0.501$

M1: main effects of genotype and environment; M2: main effects of genotype and their interactions with environment; M3: main effects of additive and dominance genetic effects and their interactions with the environment. Log-likelihoods (LLR) and *P* value (*P*val)

### Accounting for interactions between additive effects and climate variables improves genomic prediction accuracy

Variance component estimates and model comparison with loglikelihood ratio tests indicate that accounting for GxE might improve model performance, i.e. prediction accuracy (PA). To determine if accounting for GxE can also allow for more efficient evaluation of accessions in diverse samples and environments, the PA of our models is evaluated under three validation schemes: i) unobserved environments (leave-one-country-out validation), ii) unobserved germplasm, grouped based on relatedness (leave-one-genetic-cluster-out validation), and iii) unobserved germplasm, randomly grouped (leave-one-random-cluster-out validation). PA in unobserved environments (leave-one-country-out validation scheme), increased with the inclusion of GxE for TDM3, but not for SpringCover and WinterKill. PA for TDM3 increased by up to 2–4% when accounting for additive GxE, whereas for SpringCover, PA decreased by 1–4% depending on the modelling approach, while for WinterKill inclusion of additional model terms, (AxEnv, D and DxEnv), led to approx. 10% decrease in PA with the ET approach and a 21% decrease with RN (Table 4).

**Table 4:** Mean prediction accuracy and standard deviation across validation sets

		TDM3	SpringCover	WinterKill
*Leave-one-country-out*				
RN	M1	0.57 ± 0.12	0.52 ± 0.08	0.53 ± 0.11
	M2	0.61 ± 0.12	0.48 ± 0.11	0.30 ± 0.11
	M3	0.61 ± 0.11	0.48 ± 0.11	0.32 ± 0.13
ET	M1	0.57 ± 0.12	0.52 ± 0.08	0.53 ± 0.11
	M2	0.59 ± 0.11	0.52 ± 0.09	0.41 ± 0.02
	M3	0.58 ± 0.11	0.51 ± 0.09	0.43 ± 0.06
*Leave-one-genetic-cluster-out*				
RN	M1	0.03 ± 0.27	0.15 ± 0.24	0.16 ± 0.20
	M2	0.13 ± 0.27	0.37 ± 0.17	0.23 ± 0.19
	M3	0.19 ± 0.18	0.36 ± 0.17	0.26 ± 0.14
ET	M1	0.03 ± 0.27	0.16 ± 0.24	0.16 ± 0.20
	M2	0.15 ± 0.28	0.37 ± 0.17	0.25 ± 0.20
	M3	0.20 ± 0.18	0.36 ± 0.17	0.29 ± 0.14
*Leave-one-random-cluster-out*				
RN	M1	0.47 ± 0.09	0.43 ± 0.09	0.42 ± 0.24
	M2	0.50 ± 0.09	0.49 ± 0.08	0.44 ± 0.25
	M3	0.50 ± 0.09	0.48 ± 0.07	0.44 ± 0.25
ET	M1	0.47 ± 0.09	0.43 ± 0.09	0.42 ± 0.24
	M2	0.50 ± 0.10	0.49 ± 0.08	0.44 ± 0.25
	M3	0.51 ± 0.10	0.48 ± 0.07	0.44 ± 0.25

Prediction accuracies (PA) shown for the three validation schemes (Leave-one-country-out, Leave-one-genetic-cluster-out, and Leave-one-random-cluster-out) for the two modelling approaches: Reaction norm (RN) and Envirotyping (ET). M1: main effects of genotype and environment; M2: main effects of genotype and their interactions with environment; M3: main effects of additive and dominance genetic effects and their interactions with the environment

The lower PA when GxE terms were included when modelling SpringCover and WinterKill, may suggest the presence of QTL with highly environment-specific effects which would limit extrapolation of marker effects to novel environments. Results of leave-one-genetic-cluster-out validation show that for TDM3, model M1 cannot accurately extrapolate to novel germplasm, with PA effectively around zero (Table 4). However, addition of an interaction term between the additive genetic effect and environment (AxEnv) improves PA for TDM3 by up to 12%. Further extension of the model by inclusion of additional terms (D and DxEnv) further increases PA by up to 5–6%. For SpringCover and WinterKill, we observe similar improvements in PA, which increased by up to 21–22% and 7–9%, respectively, when accounting for AxEnv. These results support the presence of QTL with environment-specific effects which covary with the environmental covariates used to characterise the environments. These moderate improvements in PA further indicate that despite the accessions being genetically diverse, the models can extrapolate GxE to genetically distinct clusters of accessions.

**Fig. 3:**
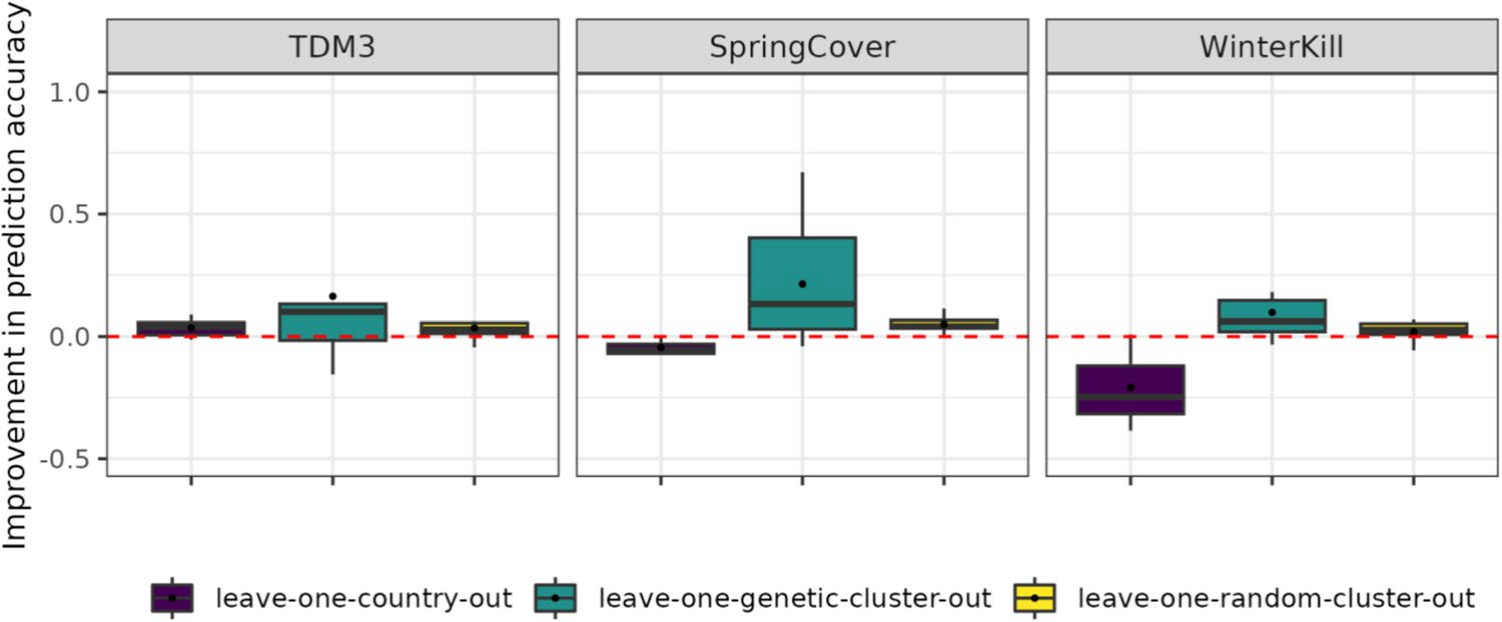
Improvement in PA when accounting for GxE under the three validation schemes. Improvement in PA from M1 to M3 shown for each validation scheme, specifically for the RN approach. The average PA for each validation scheme is denoted by a black dot

For the prediction of unobserved germplasm, randomly assigned into clusters (leave-one-random-cluster-out validation scheme), the PA of the M1 model is much improved for all traits compared to the PA observed in leave-one-genetic-cluster-out validation. However, limited improvement in PA is observed when extending the M1 with GxE terms under this validation scheme compared to the leave-one-genetic-cluster-out scheme, with PA increasing by up to 4% (TDM3), 5% (SpringCover) and 2% (WinterKill). The large improvement in PA for the leave-one-genetic-cluster-out validation compared to the leave-one-random-cluster-out validation may suggest that the additive genetic effects alone may be less useful when extrapolating to novel and genetically dissimilar germplasm, and that environment-specific marker effects may provide useful information in these scenarios. Our results show that inclusion of GxE may lead to improved PA in one validation scheme but confer no improvement or potentially reduce PA in another (Fig. 3). Neither of the two GxE modelling approaches (RN and ET) consistently outperformed the other under any of the three validation schemes. For lack of clear evidence of either modelling approach being superior, we will for downstream analyses use the RN modelling approach.

### Genomic prediction accuracy varies across countries 

**Fig. 4:**
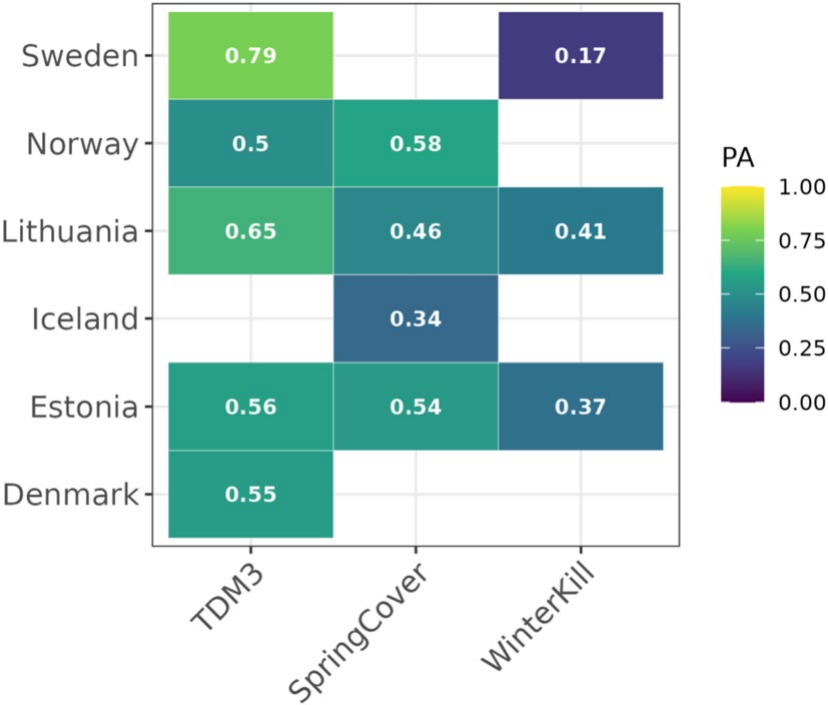
PA shown for each country in the leave-one-country-out validation scheme. GxE effects are modelled with the reaction norm (RN) approach. PA is shown for the M3 model which includes the additive and dominance genetic effects and their interaction with the environment

Environments included in this study was diverse and each uniquely challenging for the crop, which can increase the difficulty of capturing any GxE. We compare PA for each country to determine whether certain environments constitute a larger challenge for our models (Fig. 4). We note that PA for TDM3 and SpringCover is relatively stable across environments, while PA for WinterKill varies greatly across countries. These results indicate that WinterKill may be a trait which is particularly challenging to predict in novel environments. However, it is uncertain whether this is due to sparse sampling of environments (i.e. too few environments across a large area), highly environment-specific effect marker effects, and/or selected environmental variables not correlating well with phenotypic performance in specific environments.

### Genomic prediction models predict different components of phenotypic variability with varying accuracy

**Fig. 5:**
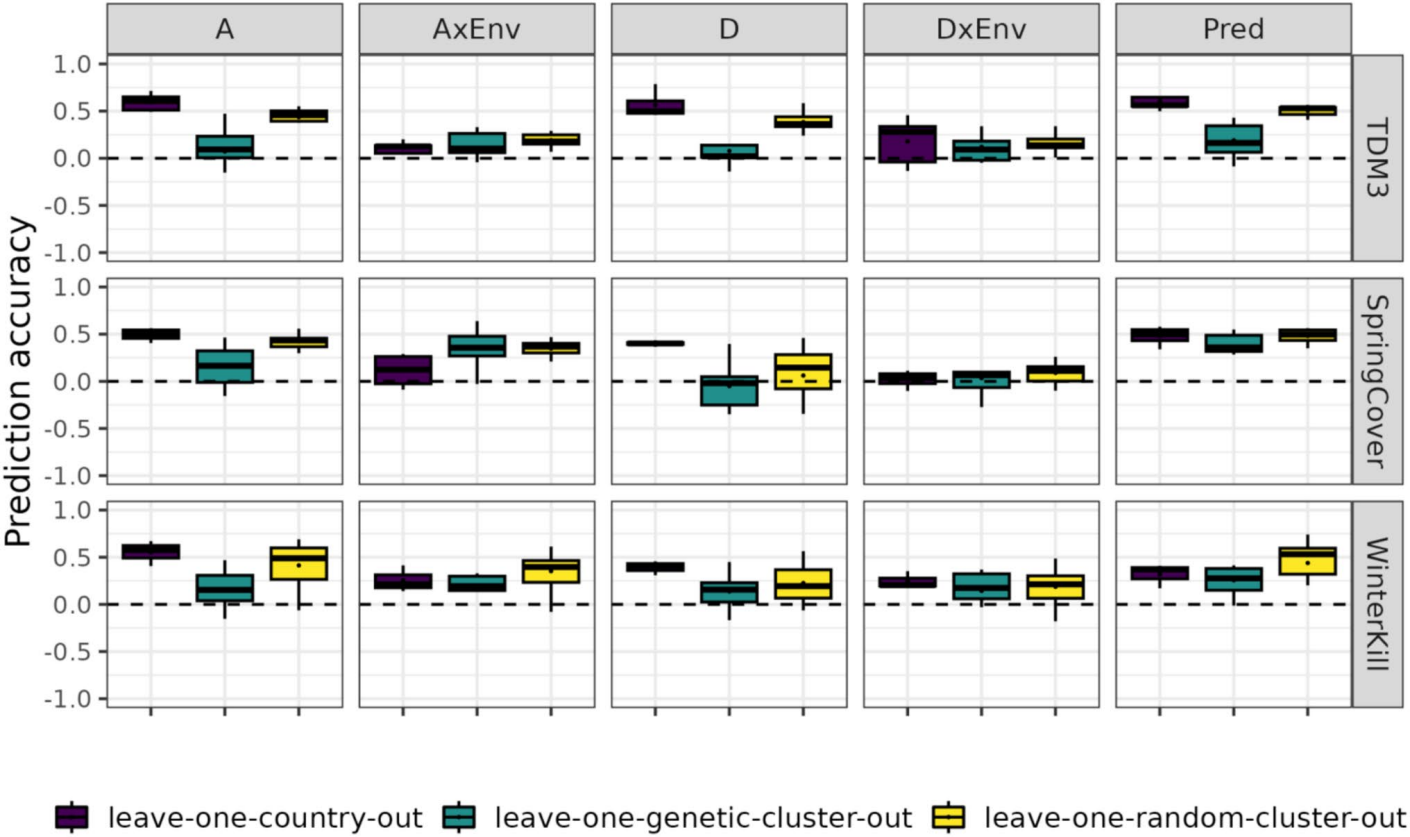
Correlation between accession means (BLUEs) and estimated component effects. Correlations in left-out folds for the three validation schemes (Leave-one-country-out scheme, Leave-one-genetic-cluster-out and Leave-one-random-cluster-out) from model M3 fitted with the reaction norm approach. The following correlations are shown Pred., Observed accession means vs. predicted accession means; A, Observed accession means vs. Additive breeding values coefficients; D, Observed accession means vs. Dominance breeding values coefficients; Env, Observed accession means vs. Environment coefficients; AxEnv, Observed accession means vs. AxEnv coefficients; DxEnv, Observed accession means vs. DxEnv coefficients

Validations show improved PA when accounting for GxE for some traits and under certain validation schemes, while for others there is a cost to including GxE (Fig. 3). Here, we investigate which model components constitute a greater challenge for our GP models. We compare the correlation between accession means ${y}_{ij}$ (for any accession *i* in environment *j*), and the estimated effects for each model component i.e. additive genetic effects (${\widehat{a}}_{i}$), dominance genetic effects (${\widehat{d}}_{i}$), interactions between additive genetic effects and environment (${\widehat{ae}}_{ij}$), and dominance genetic effects and environment (${\widehat{de}}_{ij}$). Correlations between component effects and accession means are calculated in validation sets. This analysis highlights the components which are more challenging for our models to extrapolate and/or interpolate (Fig. 5). The genetic effects (${\widehat{a}}_{i}$ and ${\widehat{d}}_{i}$) are generally well predicted for the leave-one-country-out validation scheme, with $cor\left({y}_{ij}, \widehat{{a}_{ij}}\right)$ (averaged over validation sets) ranging from 0.49 to 0.59, while $cor\left({y}_{ij}, \widehat{{d}_{ij}}\right)$ ranged from 0.39 to 0.57. When interpolating to novel germplasm, where random accessions are assigned to the validation set, the genetic effects are still reasonably well predicted, with $cor\left({y}_{ij}, \widehat{{a}_{ij}}\right)$ ranging from 0.41 to 0.44, while $\text{cor}\left({y}_{ij}, \widehat{{d}_{ij}}\right)$ ranged from 0.06 to 0.38. However, in the leave-one-genetic-cluster-out validation scheme, extrapolation of genetic effects fails, with $\text{cor}\left({y}_{ij}, \widehat{{a}_{ij}}\right)$ ranging from 0.07 to 0.16 while $\text{cor}\left({y}_{ij}, \widehat{{d}_{ij}}\right)$ ranged from -0.05 to 0.14. The leave-one-genetic-cluster-out validation scheme is furthermore characterised by large standard deviations indicating that for certain genetic clusters the genetic effects may be well predicted, whereas for other clusters $\text{cor}\left({y}_{ij}, \widehat{{a}_{ij}}\right)$ and $\text{cor}\left({y}_{ij}, \widehat{{d}_{ij}}\right)$ are either effectively zero or negatively correlated with phenotypic performance (Fig. 5). Correlations between accession means and estimated GxE show that coefficients ${\widehat{ae}}_{ij}$ and ${\widehat{de}}_{ij}$ are generally more challenging to predict under the leave-one-country-out validation scheme, as suggested by Fig. 3, which show a consistently larger cost to accounting for GxE terms in novel environments, as shown by a decrease in PA from the M1 to M3 model. Together these results underlines that the lower PA for the leave-one-genetic-cluster-out validation scheme, specifically with M1 model, compared to the other validation schemes is due to the models not being able to extrapolate genetic effects (${\widehat{a}}_{i}$ and ${\widehat{d}}_{i}$) to novel and genetically distinct germplasm. Our results further indicate that under the circumstances where the main genetic effects are not well predicted then there is a greater benefit to accounting for GxE, as shown by greater increases in PA under the leave-one-genetic-cluster-out scheme compared to the leave-one-random-cluster-out where main genetic effects are more useful to predict accession means.

### GWASs detected marginal and conditional marker effects

**Fig. 6:**
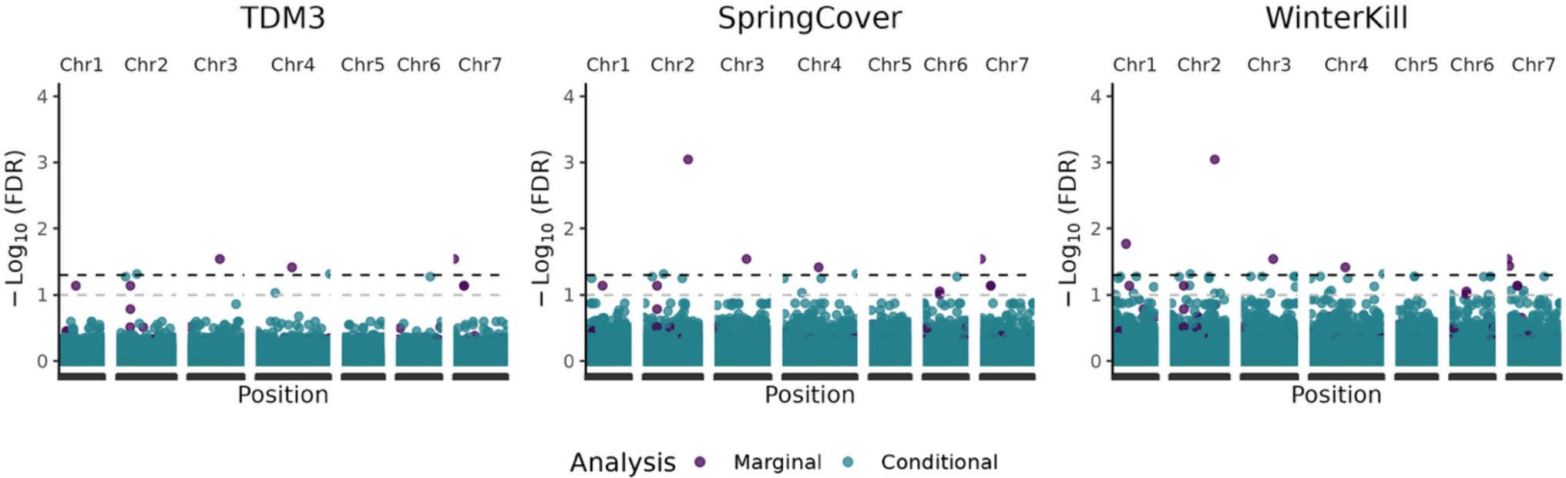
Manhattan plots from the marginal and conditional GWAS analysis. Black line denotes the significance threshold (0.05) adjusted with FDR (False-discovery rate), while grey line denotes the significance threshold (0.10) adjusted with FDR

We conducted GWASs to detect genetic variants with marginal effects, i.e. variants with environment-independent effects, and conditional effects, i.e. environment-dependent effects (Fig. 6).

**Fig. 7:**
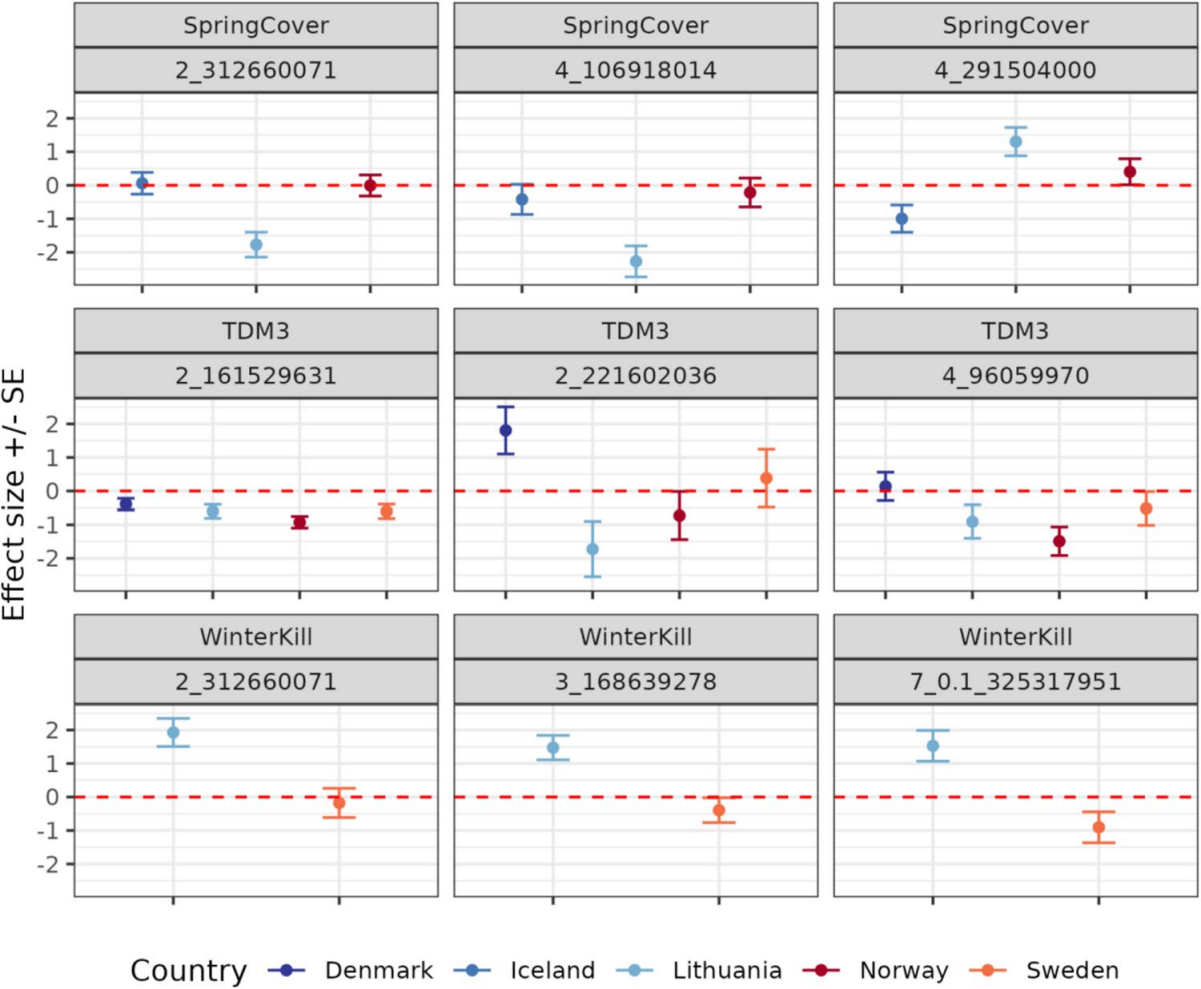
Country specific marker effects of the top three most significant SNPs. Effect estimates $\pm$ standard errors (SE) for the SNPs with the most significant P values from the conditional GWAS

Previous analyses indicated extensive additive genetic effects (Fig. 2 and Table 3) and moderate narrow-sense heritabilities (Table 2) for all traits, suggesting that a significant proportion of the phenotypic variation may be described by additive genetic variation. However, few significant QTL were detected by the marginal GWAS. These results suggest that the investigated traits are polygenic and are characterised by many small-effect QTL. Genome-wide analyses of GxE, i.e. likelihood-ratio tests (Table 3) and GP validation (Table 4), indicated the presence of environment-specific marker effects. Few conditional effects were highly significant (FDR ≤ 0.05) for TDM3. For SpringCover, there was some evidence for potential QTL (FDR ≤ 0.10) conferring a disadvantage in specific environments (Fig. 7). Similarly, for WinterKill we detected potential QTL which may confer a disadvantage in some environments but not others (Fig. 7), see Supplementary Fig. S3, and Table S2.

For both the marginal and conditional GWASs we found few significant QTL, which may in part, be due to the germplasm being genetically dissimilar as demonstrated by limited LD and rapid LD-decay (Fig. 1). Hence, only common and large-effect QTL would be detected by our models. Alternatively, it is possible that the underlying genetic architecture is highly polygenic, both in regard to the additive genetic effects and the additive GxE effects, implying that most QTL have small effects and thus challenging for the statistical models to detect. Taken together the GWAS results may indicate the presence of multiple potential QTLs, however given that the detected phenotype-genotype associations are not highly significant further validation is required.

## Discussion

### Diverse accessions contain unique genetic variation important for broadening the genetic base of perennial ryegrass

This study included a geographically and genetically diverse germplasm, to enhance the probability of including and identifying genetic variation that may confer an advantage under the environmental conditions experienced in Nordic and Baltic countries. Utilising genetically diverse accessions is favourable in prebreeding as less advanced breeding material, or potentially unselected material, may be enriched for higher genetic diversity and may contain novel adaptive variants not present in current breeding programs, which may be introgressed into more advanced breeding material to enhance phenotypic performance under biotic and abiotic stressors, as demonstrated in previous studies (Meseka et al. 2013; Singh et al. 2021). The present study sought to quantify and estimate the extent of GxE in environments that are at the limit of the species’ distribution. GxE is environment specific, hence while previous studies have investigated GxE in perennial ryegrass, most of these studies have investigated GxE in temperate regions, which are currently more suitable for production e.g. in central and southern Europe (Fois et al. 2021), Denmark (Fè et al. 2015, 2016) and Ireland (Grogan and Gilliland 2011). Consequently, the findings of these studies are presumably not indicative of the level of GxE that are present in the more extreme environments experienced in the regions investigated in the present study. GxE in environments comparable to those investigated in this study have previously been documented in perennial ryegrass, by Helgadóttir et al. (2018) who found that commercial cultivars are not widely adapted to the environmental conditions experienced across Nordic and Baltic regions, which underlines the importance of including unselected material in future breeding efforts. Additionally, the accessions included in the study have been collected from vastly different geographic origins, which is a benefit as local adaptation to some of these environments may confer an advantage in the environments tested in the current study. Hence, if we instead had included genetically similar accessions the analysis would have had to be constrained to include more closely related accessions from similar geographic backgrounds, which would have been a limitation.

### Genotype x environment interactions pose challenges in diverse samples of environments and accessions

In the current study we compared a standard reaction norm approach, as demonstrated by Jarquín et al. (2014) to a more recent envirotyping approach as demonstrated by Xu (2016) and later extended by Costa-Neto et al. (2021b, a). In accordance with the findings of previous studies we found phenotypic variation to be highly affected by GxE for both persistence traits, i.e. SpringCover and WinterKill, as well as yield. The inclusion of interactions between additive genetic effects and environments (AxEnv) led to improvements in model fit for all traits , while inclusion of dominance and dominance GxE terms further improved model fit for yield (Table 3). Previous studies, in hybrid maize, have similarly documented the importance of dominance genetic effects in regard to yield production, with (Rogers et al. 2021) demonstrating that the dominance genetic effect was a more effective predictor of yield performance than the additive genetic effect. However, when model performance was evaluated based on their ability to predict the phenotypic performance of novel germplasm, where training and validation sets were genetically more similar (leave-one-random-cluster-out), or when predicting the performance of observed germplasm in novel environments (leave-one-country-out), there were limited improvements in PA with the inclusion of GxE terms. Previous GxE studies (e.g. in wheat) experienced more marked improvements in PA when accounting for GxE, with some studies citing increases in PA by up to 82% when predicting yield performance of unobserved lines, modelled with a reaction norm approach (Jarquín et al. 2017), while (Heslot et al. 2014) reported an increase in PA of approximately 25–28% for yield production in winter wheat. It should be noted that the study by (Heslot et al. 2014) utilised crop growth models that incorporate developmental stage specific environmental covariates (stress covariates), and hence the environmental covariates included in that study was presumably more physiologically relevant than the environmental covariates included in this study. Generally, GxE studies cover less heterogeneous areas than those investigated in this study, e.g. Northern France (Jarquín et al. 2014) and France (Heslot et al. 2014). Additionally, these studies also investigated GxE across a denser spatial and temporal grid, with the study by Heslot et al. (2014) including 44 environments (location-year combinations) from 2006 to 2011, while the study by Jarquín et al. (2014) investigated 134 locations across eight years. The importance of employing a dense sampling approach is further underlined by Rogers and Holland (2022), where they noted that more sparsely sampled areas suffered higher reductions in PA as these environments were less represented by the environments included in the training set. Therefore, more effective extrapolation of GxE might be achieved with denser environmental grids even with large differences among environments. Additionally, in the current study we did not perform feature selection of the environmental covariates. However, depending on the trait and environmental conditions at the trial sites it is possible that certain environmental covariates will not covary with the scored traits, thus inclusion of these environmental covariates will introduce noise. As such, this analysis may potentially benefit from feature selection of the environmental covariates which has been shown to lead to improved PA in other studies (Montesinos-López et al. 2023). However, the dissimilarity of the environments tested in this study may challenge feature selection as the relevance of different environmental covariates may vary across sites. Further suggesting that a denser spatial grid is required for further model refinement with e.g. feature selection.

### Genetic diversity and rapid LD decay in perennial ryegrass limit the accuracy of genomic prediction

The improvements in PA, observed for certain validation schemes, are more modest than those demonstrated in previously mentioned studies. This is to be expected as the accessions are genetically diverse and include unselected material where genomic relationships between accessions are limited. This is further compounded, as we inferred genetic clusters based on genetic similarity, resulting in increased genetic dissimilarity between the accessions in the training and validation sets. Consequently, the low levels of genetic similarity between accessions in the training set and the validation set might have limited our ability to efficiently extrapolate both additive genetic effects and GxE to unobserved accessions. This is supported by the findings of Pembleton et al. (2018), which demonstrated that PA was negatively affected by the inclusion of genetically distinct subgroups of perennial ryegrass, and that PA could be enhanced by removing genetic material that is genetically distinct from the majority of the germplasm contained in the training sets. Consequently, while low genetic similarity, in general, is a challenge across species, it is possible that there is a particularly large cost to including genetically dissimilar material when performing genomic prediction in perennial ryegrass, a species characterised by rapid LD decay of less than 3 kb (Ponting et al. 2007). In our study we found rapid declines in linkage disequilibrium (LD), with LD-decay within 200 bp (Fig. 1), hence we underline the importance of including genetically similar germplasm for genomic selection in perennial ryegrass. The rapid LD-decay, observed in Fig. 1, may in part explain why relatively few small effect QTLs were detected. Although given that only a core set of 108 accessions were assessed in the different environments our ability to detect QTL with marginal effects would have been limited, while the unbalanced design, i.e. the unequal number of accessions scored in the different environments would challenge detection of QTLs with environment-specific effects. Comparison of the validation schemes, specifically leave-one-random-cluster-out and leave-one-genetic-cluster-out, shows that significantly higher PA is achievable when the genetic similarity between test and training sets are higher (leave-one-random-cluster-out). However, previous studies highlight that the presence of population and family structure in highly related populations can lead to inflated PA estimates, i.e. population structure or family structure will be more informative of phenotypic performance than the underlying marker effects (Werner et al. 2020). The higher PA for the leave-one-random-cluster-out scheme may thus be attributable to underlying population structure, which despite the diversity of the panel, remains present potentially leading to slightly inflated PA for this scheme.

### Applications for Breeders: perennial ryegrass and its future in the Northern regions

For breeders to be able to produce perennial ryegrass in more northern regions it is of importance to identify genetic material that can survive the cold and variable climates experienced in these environments. In the present study, we identified multiple genetic variants in linkage with QTL that might be associated with improved winter survival. These variants appear to have environment-independent effects across the environments where WinterKill was scored. Additionally, the presence of extensive GxE (Fig. 2 and Table 3) suggests that it may be advantageous to breed for local adaptation to specific environmental conditions or countries, as introgression of QTL with environment-specific effects may be advantageous in certain conditions while deleterious in others. Furthermore, depending on the stochasticity of the environmental stressors, at the intended production area, considerations regarding whether to select for increased GxE should be made. As environments with reliable environmental cues, e.g. seasonal changes in day-length or temperature reliably signalling future selective environments, there is an increased chance of GxE improving population persistence under stressing conditions, whereas if the environment is highly stochastic and environmental cues for acclimatisation are unreliable such interactions may be unfavourable for survival (Reed et al. 2010). Hence, if the environments at the intended production area are characterised by variable and increasingly unpredictable weather patterns it may be more beneficial to select for accessions with smaller GxE.

### Conclusions

Our study highlighted extensive GxE in perennial ryegrass in the environments experienced in Nordic and Baltic regions. We found no clear evidence for either modelling approach surpassing the other, i.e., reaction norm based on linear response to environmental variables, or envirotyping based on nonlinear responses. The environments included in this study were highly diverse, and we observed that the statistical models were challenged in modelling the GxE effect on phenotypic performance, presumably because of sparsity in the spatial grid, and while there was some benefit in accounting for GxE when predicting the performance of unobserved and genetically dissimilar germplasm, these moderate improvements in prediction were not observed for validation schemes where the additive genetic effects were most useful to predict phenotypic performance (leave-one-random-cluster out validation). Despite rapid LD-decay in our diverse panel of accessions, we detected several candidate QTL, including QTL with environment-specific effects. Together, our results suggest that there is adaptive genetic variation in the perennial ryegrass diversity panel that could be advantageous to introduce into current breeding programs. Further work investigating the extent of conditional neutrality relative to maladaptive pleiotropy in respect to local adaptation may be useful for future breeding efforts in perennial ryegrass, as this may inform breeders about the costs and benefits in selecting for increased local adaptation compared to broad adaptation.

## Data and code availability

The scripts used in this analysis are publicly available at https://github.com/TashaDear/PPP_perennial_ryegrass, and the corresponding data can be found at Zenodo, 10.5281/zenodo.16788472.

## Supplementary Information

Below is the link to the electronic supplementary material.

Supplementary file1 (DOCX 376 KB)
