# Supplementary material for: Genomic prediction of agronomic traits in perennial ryegrass (Lolium perenne L.) and genotype x environment interactions at the limit of the species distribution
Source: Theor Appl Genet. 2025 Oct 25;138(11):281. doi: 10.1007/s00122-025-05064-x (PMC12553606; doi:10.1007/s00122-025-05064-x)
Supplement: Supplementary file 1 — Supplementary file1 (DOCX 376 KB) [file 122_2025_5064_MOESM1_ESM.docx]

**Supplementary material**

| **Table S1- List of diploid perennial ryegrass accessions from the diversity panel**  Project IDs, geographic origin and accession type (breeding line, landrace, cultivar, or ecotype) for the 264 diploid accessions included in the study. | | | | | |
| --- | --- | --- | --- | --- | --- |
|  | **Accession name** | **Accession number** | **Project id** | **Country of Origin** | **Accession type** |
| *1* | 1159 | LIA368 | 40053 | Lithuania | Breeding line |
| *2* | 1159 | LIA66 | 40057 | Lithuania | Breeding line |
| *3* | 1248 | LIA365 | 40058 | Lithuania | Breeding line |
| *4* | 114-3 | 144-3 | 40128 | Estonia | Breeding line |
| *5* | 14G2000331 | 14G2000331 | 40104 | Czech Republic | Ecotype |
| *6* | 14G2000332 | 14G2000332 | 40105 | Czech Republic | Ecotype |
| *7* | 14G2000333 | 14G2000333 | 40106 | Czech Republic | Ecotype |
| *8* | 14G2000503 | 14G2000503 | 40109 | Czech Republic | Ecotype |
| *9* | 14G2000504 | 14G2000504 | 40107 | Czech Republic | Ecotype |
| *10* | 14G2000523 | 14G2000523 | 40108 | Czech Republic | Ecotype |
| *11* | 14G2000586 | 14G2000586 | 40103 | Czech Republic | Ecotype |
| *12* | 14G2000587 | 14G2000587 | 40102 | Czech Republic | Ecotype |
| *13* | 16-57-1 | NGB4262 | 40227 | Norway | Ecotype |
| *14* | 16-57-2 | NGB4263 | 40228 | Norway | Ecotype |
| *15* | 16-59-2 | NGB4264 | 40229 | Norway | Ecotype |
| *16* | 16-62-3 | NGB4267 | 40230 | Norway | Ecotype |
| *17* | 16-62-4 | NGB4268 | 40231 | Norway | Ecotype |
| *18* | 177/06 | LVA02520 | 40090 | Latvia | Breeding line |
| *19* | 264/06 | LVA02529 | 40099 | Latvia | Ecotype |
| *20* | 2903 | LIA1414 | 40071 | Lithuania | Ecotype |
| *21* | 2910 | LIA1416 | 40078 | Lithuania | Ecotype |
| *22* | 342 | PI598414 | 40188 | Turkey | Ecotype |
| *23* | 346 | PI598515 | 40189 | Turkey | Ecotype |
| *24* | 363 | PI598516 | 40190 | Turkey | Ecotype |
| *25* | 45-4 | 45-4 | 40127 | Estonia | Breeding line |
| *26* | 481 | W6-11256 | 40200 | Turkey | Ecotype |
| *27* | 512 | W6-11264 | 40201 | Turkey | Ecotype |
| *28* | 513 | PI598519 | 40191 | Turkey | Ecotype |
| *29* | 537 | LIA65 | 40080 | Lithuania | Breeding line |
| *30* | 693 | W6-11322 | 40202 | Turkey | Ecotype |
| *31* | ABY-BA10106.82 | PI619003 | 40195 | Norway | Ecotype |
| *32* | ABY-BA10109.82 | PI610803 | 40193 | Norway | Ecotype |
| *33* | ABY-BA10111.82 | PI610802 | 40192 | Norway | Ecotype |
| *34* | ABY-BA8602.00 | PI598429 | 40176 | Italy | Ecotype |
| *35* | ABY-BA8603.00 | PI598430 | 40177 | Italy | Ecotype |
| *36* | ABY-BA8604.00 | PI598431 | 40178 | Italy | Ecotype |
| *37* | ABY-BA8614.68 | PI598432 | 40179 | Italy | Ecotype |
| *38* | ABY-BA8616.00 | PI598433 | 40180 | Italy | Ecotype |
| *39* | ABY-BA8621.82 | PI598434 | 40181 | Italy | Ecotype |
| *40* | ABY-BA9080.A81 | PI598453 | 40186 | Romania | Ecotype |
| *41* | ABY-BA9088.82 | W6-9290 | 40198 | Switzerland | Ecotype |
| *42* | ABY-BA9091.72 | PI598440 | 40182 | Switzerland | Ecotype |
| *43* | ABY-BA9092.72 | PI598441 | 40183 | Switzerland | Ecotype |
| *44* | ABY-BA9097.84 | PI598443 | 40185 | Switzerland | Ecotype |
| *45* | ABY-BA9101.72 | W6-9297 | 40199 | Switzerland | Ecotype |
| *46* | ABY-BA9108.79 | PI610825 | 40194 | Switzerland | Ecotype |
| *47* | ABY-BA9792.81 | PI595043 | 40173 | UK | Ecotype |
| *48* | ABY-BA9798.82 | PI595044 | 40174 | UK | Ecotype |
| *49* | ABY-BA9803.80 | PI595047 | 40175 | UK | Ecotype |
| *50* | ABY-BA9983.81 | P1598454 | 40187 | Romania | Ecotype |
| *51* | ABY-BA9819 | BA9819 | 40356 | UK | Ecotype |
| *52* | ABY-Ba9832 | Ba9832 | 40357 | UK | Ecotype |
| *53* | ALLEGRO | NGB13338 | 40219 | Denmark | Cultivar |
| *54* | AMADO | NGB1623 | 40203 | Denmark | Cultivar |
| *55* | ANNA | POL155165 | 40397 | Poland | Cultivar |
| *56* | APUS | NGB7508 | 40238 | Sweden | Cultivar |
| *57* | ARGONA | POL155193 | 40400 | Poland | Cultivar |
| *58* | ARKE | POL155194 | 40401 | Poland | Cultivar |
| *59* | ASKELAND-16-60-1 | NGB4265 | 40232 | Norway | Ecotype |
| *60* | Abermagic | Abermagic | 40277 | UK | Cultivar |
| *61* | Algutsrum-HAJ0203 | NGB16597 | 40253 | Sweden | Ecotype |
| *62* | Aria | Vir50422 | 40146 | Sweden | Cultivar |
| *63* | Arka | 14G2000008 | 40115 | Poland | Cultivar |
| *64* | Arsenal | Arsenal | 40285 | Germany | Cultivar |
| *65* | Banat | DE161 | 40003 | Romania | Cultivar |
| *66* | Belida | NGB1633 | 40204 | Denmark | Cultivar |
| *67* | Benestad-UE1504 | NGB333 | 40254 | Sweden | Ecotype |
| *68* | BJörkeröd-PW2702 | NGB43449 | 40255 | Sweden | Ecotype |
| *69* | Ba11434 | Ba11434 | 40313 | Poland | Ecotype |
| *70* | Ba11445 | Ba11445 | 40314 | Poland | Ecotype |
| *71* | Ba11448 | Ba11448 | 40315 | Poland | Ecotype |
| *72* | Ba11451 | Ba11451 | 40317 | Poland | Ecotype |
| *73* | Ba11461 | Ba11461 | 40316 | Poland | Ecotype |
| *74* | Ba12275 | Ba12275 | 40358 | Czech Republic | Ecotype |
| *75* | Ba12276 | Ba12276 | 40359 | Czech Republic | Ecotype |
| *76* | Ba12277 | Ba12277 | 40360 | Czech Republic | - |
| *77* | Ba12947 | Ba12947 | 40319 | Hungary | - |
| *78* | Ba12948 | Ba12948 | 40320 | Hungary | - |
| *79* | Ba12949 | Ba12949 | 40321 | Hungary | - |
| *80* | Ba12950 | Ba12950 | 40322 | Hungary | - |
| *81* | Ba12951 | Ba12951 | 40323 | Hungary | - |
| *82* | Ba12952 | Ba12952 | 40324 | Hungary | - |
| *83* | Ba12953 | Ba12953 | 40325 | Hungary | - |
| *84* | Ba12954 | Ba12954 | 40326 | Hungary | - |
| *85* | Ba12955 | Ba12955 | 40327 | Hungary | - |
| *86* | Ba12956 | Ba12956 | 40328 | Hungary | - |
| *87* | Ba12957 | Ba12957 | 40329 | Hungary | - |
| *88* | Ba12958 | Ba12958 | 40330 | France | - |
| *89* | Ba12965 | Ba12965 | 40331 | France | Ecotype |
| *90* | Ba12967 | Ba12967 | 40333 | France | Ecotype |
| *91* | Ba12969 | Ba12969 | 40334 | France | Ecotype |
| *92* | Ba12970 | Ba12970 | 40335 | France | Ecotype |
| *93* | Ba12971 | Ba12971 | 40336 | France | Ecotype |
| *94* | Ba12972 | Ba12972 | 40337 | France | Ecotype |
| *95* | Ba12973 | Ba12973 | 40388 | France | Ecotype |
| *96* | Ba12974 | Ba12974 | 40339 | France | Ecotype |
| *97* | Ba12975 | Ba12975 | 40340 | France | Ecotype |
| *98* | Ba12976 | Ba12976 | 40342 | France | Ecotype |
| *99* | Ba12977 | Ba12977 | 40343 | France | Ecotype |
| *100* | Ba12978 | Ba12978 | 40344 | France | Ecotype |
| *101* | Ba12979 | Ba12979 | 40345 | France | Ecotype |
| *102* | Ba12980 | Ba12980 | 40346 | France | Ecotype |
| *103* | Ba12981 | Ba12981 | 40347 | France | Ecotype |
| *104* | Ba12982 | Ba12982 | 40348 | France | Ecotype |
| *105* | Ba12983 | Ba12983 | 40349 | France | Ecotype |
| *106* | Ba12986 | Ba12986 | 40352 | France | Ecotype |
| *107* | Ba12987 | Ba12987 | 40353 | France | Ecotype |
| *108* | Ba12988 | Ba12988 | 40354 | France | Ecotype |
| *109* | Ba12989 | Ba12989 | 4035 | France | Ecotype |
| *110* | Ba13004 | Ba13004 | 40366 | Germany | Ecotype |
| *111* | Ba13005 | Ba13005 | 40367 | Germany | Ecotype |
| *112* | Ba13006 | Ba13006 | 40368 | Germany | Ecotype |
| *113* | Ba13007 | Ba13007 | 40369 | Germany | Ecotype |
| *114* | Ba13011 | Ba13011 | 40373 | Germany | - |
| *115* | Babolnai | PI298092 | 40172 | Hungary | Landrace |
| *116* | Baca | Vir42146 | 40135 | Czechoslovakia | Cultivar |
| *117* | Barnhem | Barnherm | 40294 | Holland | Cultivar |
| *118* | Bronsyn | Bronsyn | 40288 | Holland | Cultivar |
| *119* | Burlina1 | Burlina1 | 40282 | Denmark | Cultivar |
| *120* | CHANTAL | NGB15401 | 40205 | Denmark | Cultivar |
| *121* | Calvano1 | Calvano1 | 40284 | Denmark | Cultivar |
| *122* | DASAS-TRIFOLIUM | NGB4117 | 40206 | Denmark | Cultivar |
| *123* | DE28845 | De28845 | 40031 | Germany | Ecotype |
| *124* | DE28886 | DE28846 | 40029 | Germany | Ecotype |
| *125* | DE28848 | DE28848 | 40011 | Germany | Ecotype |
| *126* | DE28849 | DE28849 | 40030 | Germany | Ecotype |
| *127* | DE28851 | DE28851 | 40018 | Germany | Ecotype |
| *128* | DE28856 | DE28856 | 40012 | Germany | Ecotype |
| *129* | DE39913 | DE39913 | 40032 | Germany | Ecotype |
| *130* | DE39932 | DE39932 | 40026 | Germany | Ecotype |
| *131* | DE33943 | DE33943 | 40017 | Germany | Ecotype |
| *132* | DE50653 | DE50653 | 40015 | Germany | Ecotype |
| *133* | DE50654 | DE50654 | 40019 | Germany | Ecotype |
| *134* | DE50667 | DE50667 | 40023 | Germany | Ecotype |
| *135* | DE50668 | DE50668 | 40027 | Germany | Ecotype |
| *136* | DE51971 | DE51971 | 40024 | Germany | Ecotype |
| *137* | DE51987 | DE51987 | 40028 | Germany | Ecotype |
| *138* | DE51990 | DE51990 | 40022 | Germany | Ecotype |
| *139* | DE54063 | DE54063 | 40040 | Romania | Ecotype |
| *140* | DE54064 | DE54064 | 40041 | Romania | Ecotype |
| *141* | DE54065 | DE54065 | 40042 | Romania | Ecotype |
| *142* | DE54084 | DE54084 | 40044 | Romania | Ecotype |
| *143* | DE54085 | DE54085 | 40045 | Romania | Ecotype |
| *144* | DE54088 | DE54088 | 40046 | Romania | Ecotype |
| *145* | DE54089 | DE54089 | 40047 | Romania | Ecotype |
| *146* | DE54090 | DE54090 | 40048 | Romania | Ecotype |
| *147* | DE54091 | DE54091 | 40049 | Romania | Ecotype |
| *148* | DE54092 | DE54092 | 40050 | Romania | Ecotype |
| *149* | DE54093 | DE54093 | 40051 | Romania | Ecotype |
| *150* | DE54456 | DE54456 | 40020 | Germany | Ecotype |
| *151* | DE54461 | DE54461 | 40016 | Germany | Ecotype |
| *152* | DE59277 | DE59277 | 40025 | Germany | Ecotype |
| *153* | DE59284 | DE59284 | 40014 | Germany | Ecotype |
| *154* | DE62301 | DE62301 | 40038 | France | Ecotype |
| *155* | DE62308 | DE62308 | 40035 | France | Ecotype |
| *156* | DE62309 | DE62309 | 40033 | France | Ecotype |
| *157* | DE62310 | DE62310 | 40034 | France | Ecotype |
| *158* | DE62312 | DE62312 | 40052 | France | Ecotype |
| *159* | DE62313 | DE62313 | 40039 | France | Ecotype |
| *160* | DE62315 | DE62315 | 40036 | France | Ecotype |
| *161* | DELTA | NGB2731 | 40239 | Sweden | Cultivar |
| *162* | E12 | NGB2594 | 40234 | Sweden | Breeding line |
| *163* | EST279 | EST279 | 40130 | Estonia | Breeding line |
| *164* | E-4-Kockoko | Vir40298 | 40134 | Poland | Cultivar |
| *165* | Fjälkinge-SB2501 | NGB4341 | 40256 | Sweden | Ecotype |
| *166* | Fure | NGB2209 | 40226 | Norway | Cultivar |
| *167* | Gothem-TL0305 | NGB1533 | 40257 | Sweden | Ecotype |
| *168* | Gunne | NHB2732 | 40240 | Sweden | Cultivar |
| *169* | Georgikon | PI632542 | 40197 | Hungary | Cultivar |
| *170* | Hagestad-PW1301 | NGB4342 | 40258 | Sweden | Ecotype |
| *171* | Häljaröd-JK3103 | NGB4345 | 40259 | Sweden | Ecotype |
| *172* | Hörsene-TL0102 | NGB1523 | 40260 | Sweden | Ecotype |
| *173* | Inka | POL155176 | 40398 | Poland | Cultivar |
| *174* | JO-0110 | DE7063 | 40001 | Finland | Breeding line |
| *175* | Jo-231 | Vir38563 | 40133 | Finland | Cultivar |
| *176* | Karcagi | PI298091 | 40171 | Hungary | Landrace |
| *177* | Karcagi | PI632510 | 40196 | Hungary | Cultivar |
| *178* | Kihelkonna-RA02066 | EST963 | 40125 | Estonia | Ecotype |
| *179* | Leningradskij-809 | DE4573 | 40010 | Soviet Union | Breeding line |
| *180* | Leninggradskii | Vir51004 | 40151 | Russia | Cultivar |
| *181* | Leningradskii-809 | Vir20258 | 40131 | Russia | Cultivar |
| *182* | Lorina | Vir49885 | 40154 | France | Cultivar |
| *183* | Markinskij-24 | DE4569 | 40007 | Soviet Union | Breeding line |
| *184* | Moskovwkij-84 | DE4570 | 40008 | Soviet Union | Breeding line |
| *185* | Martlett | Vir40889 | 40144 | Austria | Cultivar |
| *186* | NIGA | POL155230 | 40404 | Poland | Cultivar |
| *187* | NIRA | POL155192 | 40399 | Poland | Cultivar |
| *188* | NK-200 | 14G2000204 | 40119 | USA | Cultivar |
| *189* | Norlea | PI278773 | 40170 | Canda | Cultivar |
| *190* | Pasavy | DE4571 | 40006 | Soviet Union | Cultivar |
| *191* | Patora | NGB1637 | 40207 | Denmark | Cultivar |
| *192* | Pavo | NGB13331 | 40242 | Sweden | Cultivar |
| *193* | PI197270 | PI197270 | 40163 | Finland | Landrace |
| *194* | PI198070 | PI198070 | 40164 | Sweden | Landrace |
| *195* | PI204710 | PI204710 | 40165 | Turkey | Ecotype |
| *196* | PI205278 | PI205278 | 40166 | Turkey | Ecotype |
| *197* | PI272120 | PI272120 | 40167 | Poland | Ecotype |
| *198* | PI272121 | PI272121 | 40168 | Poland | - |
| *199* | PI274637 | PI274637 | 40169 | Poland | Ecotype |
| *200* | POL133323 | POL133323 | 40374 | Poland | Ecotype |
| *201* | POL133323 | POL133323 | 40374 | Ukraine | Ecotype |
| *202* | POL133324 | POL133324 | 40375 | Ukraine | Ecotype |
| *203* | POL133325 | POL133325 | 40376 | Slovakia | Ecotype |
| *204* | POL133326 | POL133326 | 40377 | Slovakia | Ecotype |
| *205* | POL133327 | POL133327 | 40378 | Ukraine | Ecotype |
| *206* | POL133328 | POL133328 | 40379 | Poland | - |
| *207* | POL133398 | POL133398 | 40380 | Poland | - |
| *208* | POL133399 | POL133399 | 40381 | Poland | - |
| *209* | POL133400 | POL133400 | 40382 | Poland | - |
| *210* | POL133401 | POL133401 | 40383 | Poland | Ecotype |
| *211* | POL155145 | POL155145 | 40384 | Poland | Ecotype |
| *212* | POL155147 | POL155147 | 40385 | Poland | Ecotype |
| *213* | POL155148 | POL155148 | 40386 | Poland | Ecotype |
| *214* | POL155150 | POL155150 | 40387 | Poland | Ecotype |
| *215* | POL155151 | POL155151 | 40388 | Poland | Ecotype |
| *216* | POL155152 | POL155152 | 40389 | Poland | Ecotype |
| *217* | POL155153 | POL155153 | 40390 | Poland | Ecotype |
| *218* | POL155154 | POL155154 | 40391 | Poland | Ecotype |
| *219* | POL155155 | POL155155 | 40392 | Poland | Ecotype |
| *220* | POL155158 | POL155158 | 40393 | Poland | Ecotype |
| *221* | POL155159 | POL155159 | 40394 | Poland | Ecotype |
| *222* | POL155160 | POL155160 | 40395 | Poland | Ecotype |
| *223* | Presto-Pajbjerg | NGB8378 | 40396 | Denmark | Cultivar |
| *224* | Priekulskij-59 | DE4572 | 40209 | Soviet Union | Breeding line |
| *226* | Perma | Vir36944 | 40116 | Holland | Cultivar |
| *227* | Portrush | Portrush | 40153 | UK | Cultivar |
| *228* | Portstewart | Portstewart | 40297 | UK | Cultivar |
| *229* | Prosperowo/BY | Vir47191 | 40296 | Poland | Cultivar |
| *230* | Pürksi-Karja-RA0271 | EST968 | 40139 | Estonia | Ecotype |
| *231* | RIIKKA | NGB8417 | 20221 | Finland | Cultivar |
| *232* | RONJA | BGB13330 | 40243 | Sweden | Cultivar |
| *233* | RONJA | NGB14164 | 40244 | Sweden | Cultivar |
| *234* | Raidi | EST49 | 40123 | Estonia | Cultivar |
| *235* | SERVO | NGB2444 | 40245 | Sweden | Cultivar |
| *236* | SILDIG-DAENO III | NGB11675 | 40218 | Denmark | Cultivar |
| *237* | SILDIG HUNSBALLE | NGB1641 | 40211 | Denmark | Cultivar |
| *238* | SIVERSKIJ-809 | DE22479 | 40004 | Soviet Union | Cultivar |
| *239* | SVEA | NGB2730 | 40246 | Sweden | Cultivar |
| *240* | SVENSK-01408-NO | NGB11673 | 40235 | Sweden | Breeding line |
| *241* | SW-E39 | NGB13887 | 40236 | Sweden | Breeding line |
| *242* | SW-E50 | NGB13888 | 40237 | Sweden | Breeding line |
| *243* | TACA-Trifolium | NGB1642 | 40212 | Denmark | Cultivar |
| *244* | TAYA | NGB13328 | 40213 | Denmark | Cultivar |
| *245* | TOFTA-TL0102 | NGB1568 | 40261 | Sweden | Ecotype |
| *246* | TRANI | NHB4092 | 40215 | Denmark | Cultivar |
| *247* | Toronto | Toronto | 40287 | Germany | Cultivar |
| *248* | VALINGE | NGB2602 | 40220 | Finland | Cultivar |
| *249* | VALINGE | NGB2590 | 40251 | Sweden | Landrace |
| *250* | VALINGE | NGB4090 | 50252 | Sweden | Landrace |
| *251* | VEJA | DE22481 | 40005 | Soviet Union | Cultivar |
| *252* | VERNA-PAJBJERG | NGB1647 | 40217 | Denmark | Cultivar |
| *253* | VIKTORIA | NGB6267 | 40248 | Sweden | Landrace |
| *254* | VIRIS | NGB2360 | 40249 | Sweden | Landrace |
| *255* | VIVA | NGB2729 | 40250 | Sweden | Cultivar |
| *256* | VOLL-16-62-2 | NGB4266 | 40233 | Norway | Cultivar |
| *257* | Vir37860 | Vir37860 | 40132 | Romania | Cultivar |
| *258* | Vir47200 | Vir47200 | 40155 | Poland | Ecotype |
| *259* | Vir47226 | Vir47226 | 40142 | Poland | Ecotype |
| *260* | Vir49962 | Vir49962 | 40145 | Moldova | Ecotype |
| *261* | Vir51517 | Vir51517 | 40159 | USA | - |
| *262* | Vir51519 | Vir51519 | 40161 | Russia | Ecotype |
| *263* | Vir51520 | Vir51520 | 40162 | Ukraine | Ecotype |
| *264* | WIR20258 | NGB4261 | 40223 | Norway | Breeding line |
| *265* | Yomulin/PT | Vir47196 | 40140 | Poland | Cultivar |

| **Table S2-List of environmental covariates used to construct the environmental similarity matrices** | |
| --- | --- |
| **ID** | **Environmental covariate** |
| **ALLSKY_SFS_SW_DWN** | All sky insolation incident on a horizontal surface |
| **ALLSKY_SFC_LW_DWN** | Thermal infrared longwave radiative flux |
| **ETP** | Evatransportation |
| **FRUE** | Effect of temperature and radiation use efficiency |
| **n** | Duration of sunshine hours |
| **N** | Daylight hours |
| **PETP** | Atmospheric water deficit |
| **PRECTOT** | Rainfall precipitation |
| **RH2M** | Relative air humidity at 2 Meters |
| **RTA** | Global Solar Radiation based on latitude and Julian day |
| **SPV** | Slope of saturation vapour pressure curve |
| **T2MDEW** | Dew-point temperature at 2 Meters |
| **T2M** | Temperature at 2 Meters |
| **T2M_MIN** | Minimum air temperature at 2 Meters |
| **T2M_MAX** | Maximum air temperature at 2 Meters |
| **T2M_RANGE** | Temperature range at 2 Meters |
| **VPD** | Vapour pressure deficiency |
| **WS2M** | Wind speed at 2 Meters |
|  | |

| **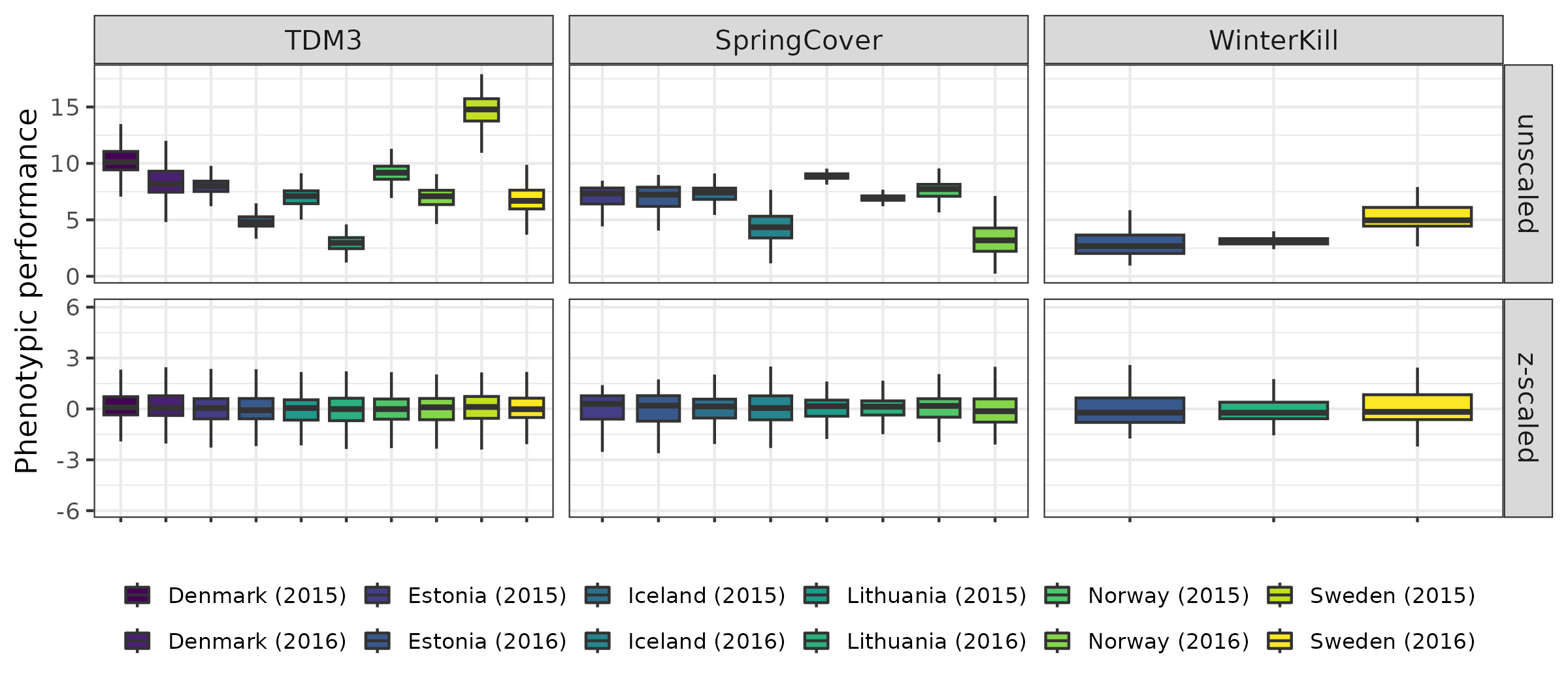** |
| --- |
| **Figure S1- Environment-specific distribution of phenotypes prior and after scaling**  Distribution of phenotypes, i.e. after adjusting for micro-environmental effects shown prior to centering and scaling (unscaled), and after z-scaling shown for each environment where the traits have been scored. |

| **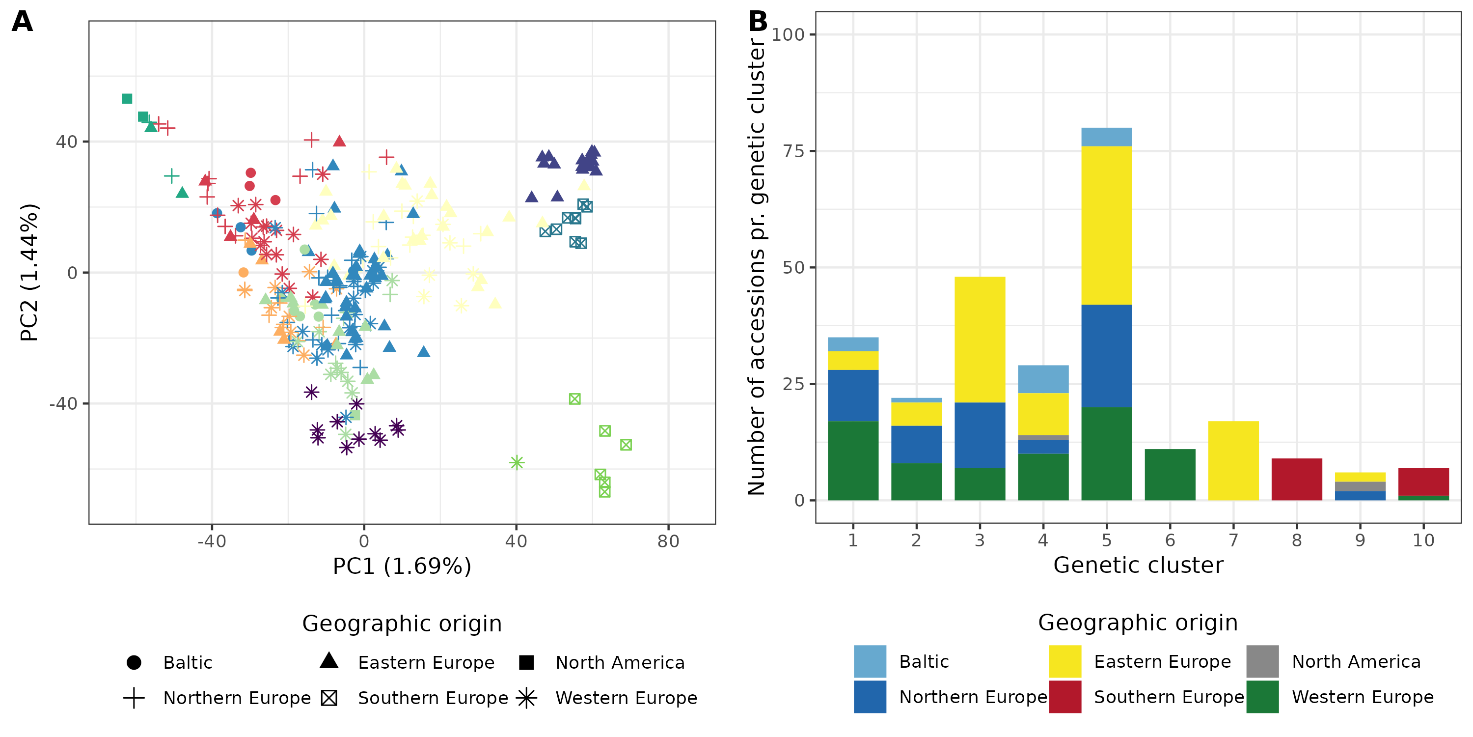** |
| --- |
| **Figure S2- PCA plot of the 264 perennial ryegrass accessions based on their additive allele dosage and geographic origin**  A)PCA plot showing the assignment of the 10 genetic clusters and annotated with geographic origin. B) Barplot showing the number of accessions assigned to each genetic cluster, along with the proportion of each genetic cluster originating from the different geographic origin. |

| 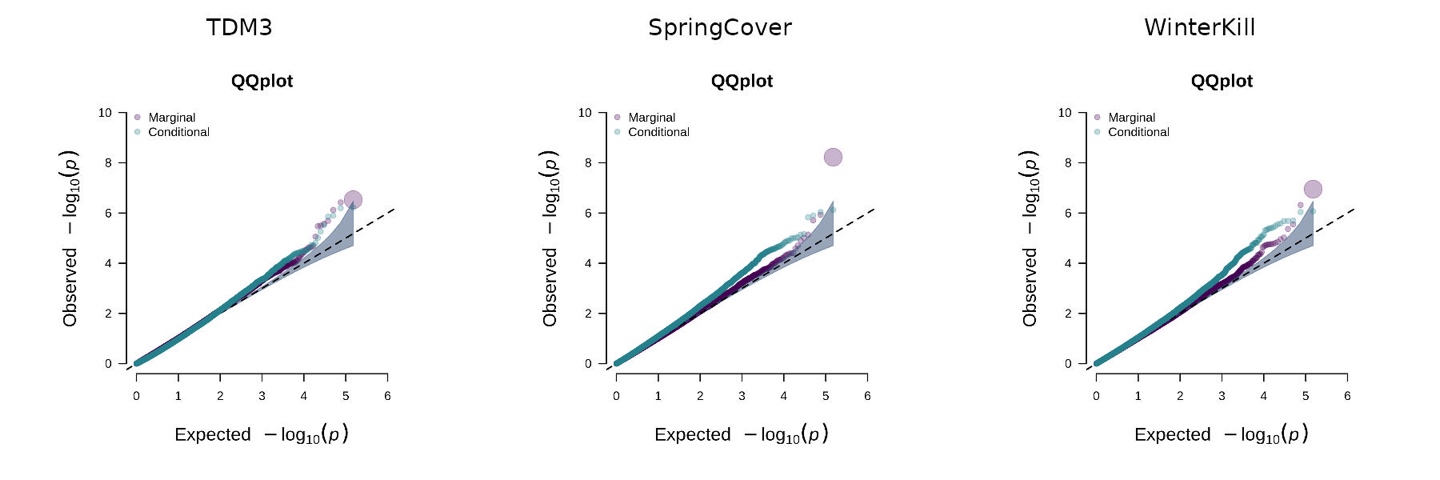 |
| --- |
| **Figure S3- QQplots for the p-values from the GWAS analyses (marginal and conditional GWASs)**  P-values largely follow the diagonal, which suggests that our models effectively control for confounding factors like population stratification. |

| **Table S2- P-values for SNPs below the genomewide threshold (FDR)** | | | | | |
| --- | --- | --- | --- | --- | --- |
| **Trait** | **Analysis** | **Chr** | **Position** | **P-value** | **FDR** |
| SpringCover | Conditional | 1 | 137827458 | 1.49$\cdot$10^-6^ | 0.056 |
| SpringCover | Conditional | 2 | 312660071 | 7.54$\cdot$10^-7^ | 0.056 |
| SpringCover | Conditional | 4 | 106918014 | 1.25$\cdot$10^-6^ | 0.056 |
| SpringCover | Conditional | 4 | 291504000 | 9.14$\cdot$10^-7^ | 0.056 |
| SpringCover | Marginal | 2 | 340594974 | 5.99$\cdot$10^-9^ | 0.0009 |
| SpringCover | Marginal | 6 | 198771042 | 1.95$\cdot$10^-6^ | 0.098 |
| SpringCover | Marginal | 6 | 198771051 | 1.17$\cdot$10^-6^ | 0.088 |
| TDM3 | Conditional | 2 | 161529631 | 1.26$\cdot$10^-6^ | 0.053 |
| TDM3 | Conditional | 2 | 221602036 | 6.02$\cdot$10^-7^ | 0.048 |
| TDM3 | Conditional | 4 | 96059970 | 6.38$\cdot$10^-7^ | 0.048 |
| TDM3 | Conditional | 4 | 189930020 | 3.07$\cdot$10^-6^ | 0.092 |
| TDM3 | Conditional | 6 | 35366913 | 1.40$\cdot$10^-6^ | 0.052 |
| TDM3 | Marginal | 1 | 193676613 | 3.36$\cdot$10^-6^ | 0.073 |
| TDM3 | Marginal | 2 | 188828089 | 2.68$\cdot$10^-6^ | 0.073 |
| TDM3 | Marginal | 3 | 306736484 | 2.96$\cdot$10^-7^ | 0.029 |
| TDM3 | Marginal | 4 | 277056746 | 7.57$\cdot$10^-7^ | 0.038 |
| TDM3 | Marginal | 7 | 103894370 | 3.79$\cdot$10^-7^ | 0.029 |
| TDM3 | Marginal | 7 | 159935238 | 3.1$6\cdot$10^-6^ | 0.073 |
| TDM3 | Marginal | 7 | 159935246 | 2.05$\cdot$10^-6^ | 0.073 |
| WinterKill | Conditional | 1 | 12416317 | 1.87$\cdot$10^-5^ | 0.098 |
| WinterKill | Conditional | 1 | 74714761 | 8.93$\cdot$10^-6^ | 0.075 |
| WinterKill | Conditional | 1 | 149556644 | 3.33$\cdot$10^-6^ | 0.053 |
| WinterKill | Conditional | 1 | 212715132 | 1.55$\cdot$10^-5^ | 0.094 |
| WinterKill | Conditional | 1 | 227248048 | 2.11$\cdot$10^-6^ | 0.053 |
| WinterKill | Conditional | 2 | 57085778 | 1.55$\cdot$10^-5^ | 0.094 |
| WinterKill | Conditional | 2 | 218558177 | 7.14$\cdot$10^-6^ | 0.072 |
| WinterKill | Conditional | 2 | 312660071 | 8.55$\cdot$10^-7^ | 0.052 |
| WinterKill | Conditional | 2 | 340594974 | 1.56$\cdot$10^-5^ | 0.094 |
| WinterKill | Conditional | 3 | 74496447 | 4.86$\cdot$10^-6^ | 0.053 |
| WinterKill | Conditional | 3 | 83644758 | 4.38$\cdot$10^-6^ | 0.053 |
| WinterKill | Conditional | 3 | 83644766 | 4.73$\cdot$10^-6^ | 0.053 |
| WinterKill | Conditional | 3 | 83644804 | 9.00$\cdot$10^-6^ | 0.076 |
| WinterKill | Conditional | 3 | 168639278 | 9.14$\cdot$10^-7^ | 0.053 |
| WinterKill | Conditional | 3 | 249015291 | 4.02$\cdot$10^-6^ | 0.053 |
| WinterKill | Conditional | 3 | 293400020 | 1.5$3\cdot$10^-5^ | 0.094 |
| WinterKill | Conditional | 4 | 53655409 | 7.86$\cdot$10^-6^ | 0.074 |
| WinterKill | Conditional | 4 | 152399748 | $2.79\cdot$10^-6^ | 0.053 |
| WinterKill | Conditional | 4 | 201753647 | 1.15$\cdot$10^-5^ | 0.086 |
| WinterKill | Conditional | 5 | 203021725 | 2.09$\cdot$10^-6^ | 0.053 |
| WinterKill | Conditional | 5 | 203021751 | 2.62$\cdot$10^-6^ | 0.053 |
| WinterKill | Conditional | 6 | 75411051 | 3.73$\cdot$10^-6^ | 0.053 |
| WinterKill | Conditional | 6 | 75411052 | 4.01$\cdot$10^-6^ | 0.053 |
| WinterKill | Conditional | 6 | 64101962 | 1.90$\cdot$10^-5^ | 0.098 |
| WinterKill | Conditional | 6 | 107590067 | 1.70$\cdot$10^-5^ | 0.099 |
| WinterKill | Conditional | 7 | 124140775 | 1.18$\cdot$10^-5^ | 0.086 |
| WinterKill | Conditional | 7 | 203027370 | 1.87$\cdot$10^-5^ | 0.099 |
| WinterKill | Conditional | 7 | 203277106 | 1.19$\cdot$10^-5^ | 0.086 |
| WinterKill | Conditional | 7 | 325317951 | 2.00$\cdot$10^-6^ | 0.053 |
| WinterKill | Marginal | 1 | 176887443 | 1.12$\cdot$10^-7^ | 0.017 |
| WinterKill | Marginal | 7 | 114853062 | 4.82$\cdot$10^-7^ | 0.036 |
|  | | | | | |
